# Assessing the impact of glazing and window shade systems on view clarity

**DOI:** 10.1038/s41598-024-69026-x

**Published:** 2024-08-08

**Authors:** Won Hee Ko, Isabel Burgess, Stefano Schiavon, Susana T. L. Chung, Piers MacNaughton, Chai Yoon Um

**Affiliations:** 1 Hillier College of Architecture and Design, New Jersey Institute of Technology, Newark, NJ USA; 2 Center for the Built Environment, University of California, Berkeley, CA USA; 3 School of Optometry, University of California, Berkeley, CA USA; 4 View Inc., Milpitas, CA USA

**Keywords:** Civil engineering, Engineering

## Abstract

Windows provide access to daylight and outdoor views, influencing building design. Various glazing and window shade materials are used to mitigate glare, overheating and privacy issues, and they affect view clarity. Among them, we evaluated the effect of window films, electrochromic (EC) glass, and fabric shades on view clarity. We conducted an experiment with 50 participants using visual tests adapted from clinical vision tests (visual acuity, contrast sensitivity, color sensitivity) and images displayed on a computer monitor in a controlled laboratory. Window films and EC glass tints outperformed fabric shades in visual acuity, contrast sensitivity and view satisfaction with the exception of the darkest EC tint state and dark grey VLT 3% shade for color sensitivity and view satisfaction. The EC tints pose internal reflection issues and fabric shades are preferred for visual privacy. Window films and EC glass hinder participants’ blue–green color discrimination while fabric shades also decrease red–yellow color discrimination. Visual acuity predicts view satisfaction and contrast sensitivity is the strongest predictor for visual privacy. Generally, higher visible light transmittance and lower solar reflectance (darker color) enhance human visual performance. The proposed workflow provides an experimental procedure, identifies the primary variables and establishes a predictive framework for assessing view clarity of fenestration.

## Introduction

Glazing and window shade materials are used to reduce the risks of glare, overheating^1,2^, and privacy issues^3,4^. However, they can also block or distort the window view^5,6^. In building science research, the effect on building occupant’s perception of outdoor view is under-studied despite the positive impacts of window view on occupants^7^. These potential impacts include improved health, well-being^8^, emotion^9^, cognitive performance^9–11^, and stress recovery^12^.

Three primary variables determine window view quality: content, access, and clarity^13^. Clarity addresses how clearly the visual content in the view can be seen by the occupant. It encompasses the visual obstructions present at the window—before, after, or inside the glazing layer(s)—and the temporal characteristics impacted by, or responding to, external stimuli (e.g., direct sun)^13^. Clarity is directly influenced by the properties of glazing and window shades, as well as by temporal characteristics that impacted by, or responding to, external stimuli (e.g., daylight intensity and solar angles)^14^. Additionally, window design, the content of outdoor views, and observer characteristics (e.g., ocular performance, color vision) affect the observer’s ability to see the outdoor views through a fenestration.

While a direct link exists between fenestration design and clarity, few standards address the topic. Daylighting standards and green certification systems, such as European Daylighting Standard EN17037 and LEED v4, lack systemic guidance on considering clarity of glazing and window shade materials in buildings. Rather, they require clear, neutrally colored view windows, free from distortion caused by frits, fibers, patterned glazing, or added tints^15,16^. Similarly, ASHRAE 189.1^17^ defines view fenestration as undiffused glazing with a haze value less than 3%^18^. European standard EN 14501^19^ evaluates the effect of blinds and shutters on exterior view based on two transmittance parameters. However, this standard is not applicable for assessing the diverse visual effects from other shade materials on occupants.

Changes in types of shade materials and their optical properties can lead to different occupant view perceptions. Some researchers have investigated the effect of roller shades (e.g., fabric shades) on clarity: the View Clarity Index^20^ and its revised version^21^ were developed to evaluate clarity of view through solar shading fabrics. In these studies, the authors demonstrated that some optical properties of the fabric such as visible light transmittance (VLT) and the angular variation of direct-direct (directional radiation to directional radiation) and direct-diffuse (directional radiation to diffused radiation) transmittance of different fabrics influences view clarity through the shade. Additionally, there are comparison studies considering fabric shade effects on glare and color fidelity characterization^22^ as well as on daylight availability^23^. These studies show that darker colored fabrics can lead to a higher clarity of view and better color perception, and for a given color, a higher openness factor will lead to increased clarity. Specular materials, such as tints, films, and electrochromic glass (EC), are also commonly used in facade design and their effect on view perception requires further investigation. Specular materials mainly shift brightness, reflectivity, and color perception while maintaining transparency through the materials^24–26^. The tint color of glazing affects both the visual performance of people indoors and their perception of outdoor content. Some researchers have demonstrated that blue-tinted spectacles reduce visual performance, while grey spectacles offer some improvement^26^. Other study have found that yellow tints enhance the contrast between bright content and a blue background^27^.

The temporal characteristics of daylight and view content are difficult to control and thus are typical problems for daylight and window research. For example, lighting conditions affect view clarity and the effect of view clarity on view perception may vary depending on the view content. Previous studies on view clarity were mostly conducted under daylight condition with an external window where the lighting conditions and view content cannot be controlled, which could lead a large amount of variance in the recorded data^20–22^. Though less realistic, conducting an experiment in a controlled laboratory environment minimizes confounding factors associated with varying daylight conditions, such as the effect of light levels that are conducive to visual perception and comfort, which may in turn influence human visual performance and view satisfaction. Moreover, the controlled laboratory setting allows the research team to have the ability to test how view clarity through different glazing and shade materials affect how people perceive the different view content.

### Problem statement

Glazing and shading system properties and their design parameters affect view clarity but quantification of these effects is limited. Furthermore, there is not a systematic assessment method that can be applied to a wide range of fenestration materials to understand their impact on clarity levels and resulting occupant view quality. Therefore, this study aims to develop an experimental procedure for assessing view clarity, identify the primary variables, and propose a prediction framework. We propose a replicable research method to evaluate how view clarity in fabric shades, window films, and EC glass affects occupants’ visual performance and its consequential effect on view satisfaction. To achieve this aim, we conducted a human-subject study in a controlled laboratory environment.

## Methods

### Experimental design

**Figure 1:**
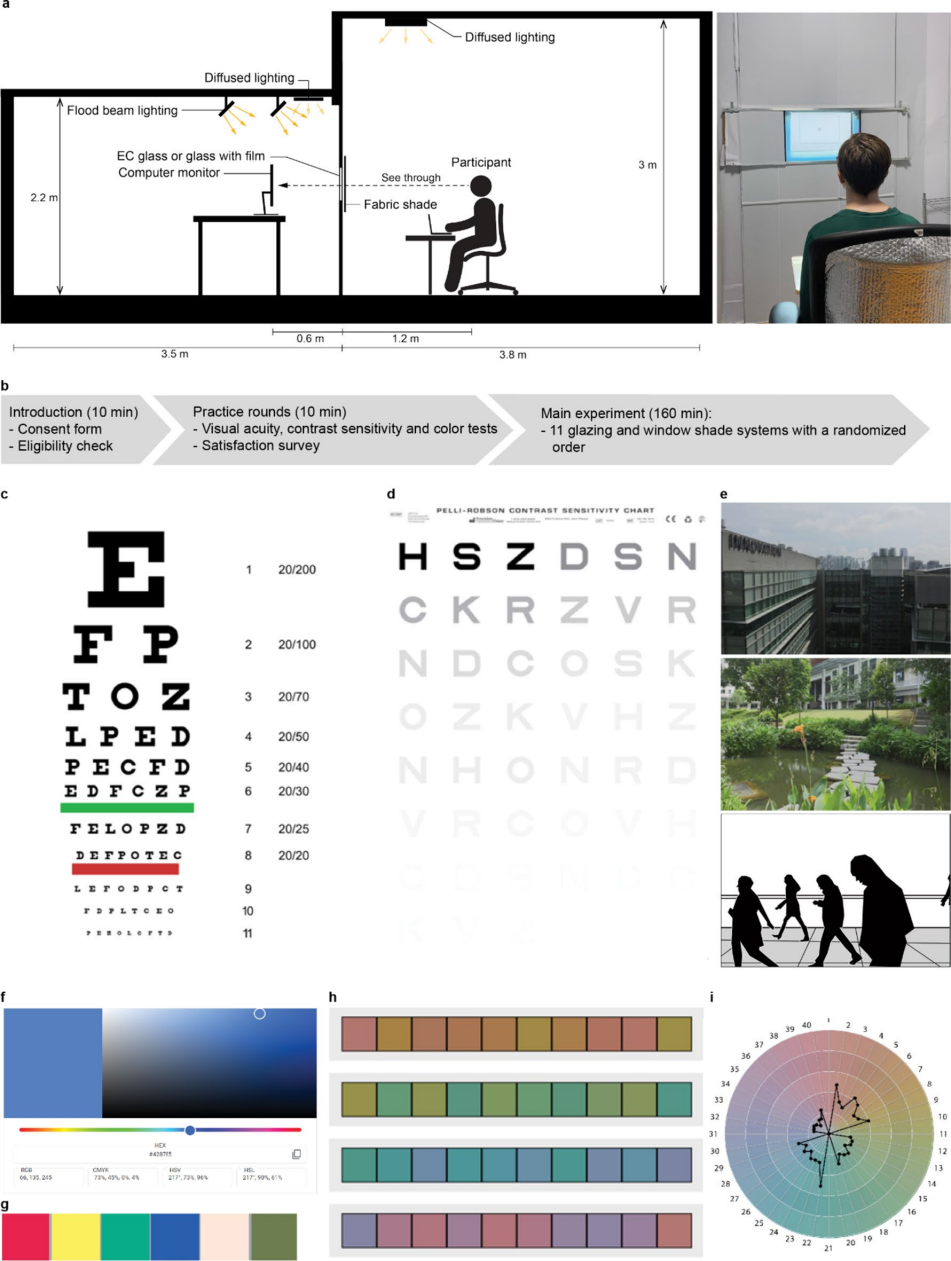
(**a**) Diagram of test room setup and the “interior” with a seated participant (participant consented to have this image published in an online open access publication); (**b**) Experimental procedure; (**c**) The Snellen Chart for visual acuity; (**d**) The Pelli-Robson contrast sensitivity chart; (**e**) View Satisfaction images (“Building”, “Pond”, “People”). The “People” image was recreated from a real photograph depicting a bustling street, transformed into line drawings to adhere to copyright regulations.; (**f**) Google color picker; g. CIE TCS 9–14 color swatches; (**h**) Color Challenge and Hue Test; (**i**) Circle graph.

We conducted a randomized within-subject experiment. Each subject participated in a three-hour long experimental session that included 11 glazing and shade material conditions (Table 1). For each material condition, we recorded human visual performance on multiple assessments and view satisfaction (Fig. 1). To avoid an order effect, we used a modified version of Microsoft Excel’s randomization function to create a random order sequence for the 11 different conditions for each participant. The experiments took place between April and August 2022.

**Table 1:** Glazing and window shade materials by type.

Type	Description	Name**	VLT	VLT′	OF*	RS	RS′	Appearance
Films	Film with clear glass	Film VLT 5%	9	5.0		58	21	
		Film VLT 3%	6	3.2		55	19	
EC glass	Tint level 2	EC-2 VLT 31%	31			10		
	Tint level 3	EC-3 VLT 6%	6			10		
	Tint level 4	EC-4 VLT 1%	1			31		
Fabric shades	Fabric shade with clear glass	Light Grey VLT 6%	11	5.9	9.6	31	11	
		Dark Grey VLT 3%	6	3.2	4	9	3.0	
		Medium Grey VLT 3%	5	2.7	4.5	25	8.5	
		Black VLT 2%	4	2.2	3.2	5	1.7	
		Black VLT 1%	2	1.1	1.8	5	1.7	
		Dark Grey VLT 1%	1	0.5	1	10	3.4	

*Measured OF values applicable only to fabric shades within 1% tolerance of the manufacturer’s target OF.

**Manufacturer product names provided in Appendix B in Supplementary Information online.

### Participants

50 participants (26 females, 22 males, and 2 non-binary) took part in the experiment. Researchers sent email invitations to several departments at the University of California, Berkeley, and used an online participant recruitment platform for Berkeley students to enroll subjects into the study. Pre-screening criteria included: age between 18 and 40 years, not pregnant, no self-reported vision impairments (e.g., color blindness, eye disorders), and no adult attention-deficit/hyperactivity disorder (ADHD). All participants provided informed consent, and each participant was compensated $60 for 3 h of the in-person lab experiment. Participant median age was 23 years, 52% wore corrective lenses, and median reported sensitivity to lighting and visual conditions was 3 on a scale from 0—much lower to 5—much higher.

### Test room set-up and equipment

Experiments were conducted at the University of California, Berkeley in a physical lab space with an internal window and a computer screen “view” (Fig. 1a). We placed an iMac 27-inch 3.4 GHz Quad-core i7 Apple desktop monitor 0.6 m behind a 0.5 × 0.4 m window configuration and placed a desk and office chair on the other side of the window so that a seated participant had their eyes at a 1.2 m distance from the middle point of the glass. Participants adjusted their seat height so their eye level aligned with the center of the window at 1.2 m above the floor.

One circular diffuse fixture provided light in the space above the participant, referred to as the “interior” (Fig. 1a). On the other side of the window, the “exterior”, six 10,000 lm (6000 K CCT) flood lights were mounted above the monitor and angled at a 45-degree angle from the ceiling towards the window to simulate directional light hitting on the window surface. The angle was chosen to avoid light sources shown in the subjects’ field of view, aligning with other studies on view clarity^20,22^. This approach minimizes the impact of glare on the perception of clarity while maximizing vertical illuminance from the lighting sources. One diffuse fixture was placed on the ceiling between the monitor and the window. Both diffuse and flood lighting fixtures did not create any direct reflection or visible inhomogeneity to the screen surface. The vertical illuminance value was approximately 10,300 lx at the center of glass facing towards the monitor on the “exterior”, which is comparable to the low-end range of the hourly outdoor illuminance level, and a horizontal illuminance baseline value was approximately 800 lx at desk level (0.8 m from floor, 0.9 m horizontally back from window on the “interior” participant side) for the unglazed opening before glazing and shading materials were installed. See Appendix A in Supplementary Information online for full illuminance and luminance values.

### Glazing and window shade materials

We assessed two commonly used shade material types in buildings: specular materials and fabric shades. Multiple optical properties of these materials may impact view clarity. VLT and solar reflectance (RS), the ratio between the solar energy globally reflected by the material and total incident solar energy are the two major manufacturer-provided variables for both specular materials and fabric shades. For fabric shades, openness factor (OF, perforation rate of the fabric) and color, which is related to the reflectance of the material, are also primary variables in determining the view clarity of semi-transparent shade materials^20^. The angular variation of direct-direct (directional radiation to directional radiation) and direct-diffuse (directional radiation to diffused radiation) transmittance of different fabrics can also be primary variables that affect view perception of blinds and shutters, as identified in the European standard EN 14501^19^ and literature on fabric shades^21^. However, angular variation of direct-direct and direct-diffuse transmittance values are often not provided by the manufacturer and are expensive to measure in a lab. Therefore, we selected films, EC glass, and fabric shades that have comparable and commercially listed optical properties, especially VLT, with variations of RS and OF.

EC glazing uses a conductive film applied to the inside surface of an insulated glass unit to change the VLT of the glass depending on the current that is passed through the film, allowing it to change dynamically depending on temporal and spatial solar conditions or based on user commands. For the cases of window films and fabric shades, a clear glass material is necessary to overlay with, therefore we considered VLT′ value while selecting the materials. VLT′ value is calculated by multiplying the VLT values of a film (or a fabric) and the clear glass. Even if the exact overall VLT values may slightly differ, it is acceptable to compare and select window and shade materials based on VLT′. RS′ value is calculated as same as VLT′.

While selecting materials, we used mainly three tint levels of one EC glazing products, and fabric shade and films with comparable VLT′ values as the basis: 31%, 5–6%, 2–3% and 1%. Appendix B in Supplementary Information online includes the product information for each glazing and window shade material, according to the VLT ranges. Table 1 summarizes the information of selected materials by type. We also have no glass and clear glass cases as practice rounds and baseline information but these are not included in the result section for brevity and discussed further in Appendix B in Supplementary Information online.

### Procedure

The Committee for the Protection of Human Subjects at Berkeley reviewed and approved the study protocol (2022-02-15020). One participant took part in the study at a time. Each time slot lasted 3 h and occurred between 9 am and 6:30 pm. Figure 1b describes the experimental procedure. Once the participant arrived, researchers introduced the study and had the participant read and sign a consent form.

Before commencing the experiment, participants needed to pass diagnostic color deficiency and visual acuity tests to ensure baseline eye functioning. Researchers ran the color vision test^28^ developed based on the Ishihara Test^29^ with no glazing installed in the experimental setup to check for normal color vision. Participants needed to correctly identify the number in 7 out of 12 Ishihara number test plates shown on the computer monitor to pass the test and participate in the experiment. Participants then read the line of letters on an acuity test that corresponded to the standard acuity of 20/20 at a standard distance. If they needed to come closer to the acuity chart to read the line (Fig. 1c), they failed the test. All participants passed the tests and no one was eliminated. After this, researchers ran the contrast sensitivity, color matching, and color arrangement tests (Fig. 1d, f, h) all without glazing installed to allow the participants to familiarize themselves with the test procedures.

To familiarize participants with the different types of glazing and shading and the satisfaction survey procedure, they saw three scene images (gray building, green pond, and street view with people, Fig. 1e), through a clear glass, a fabric case and an EC glass case, and filled out a practice satisfaction survey on a provided laptop. Participants swiveled in their chair to face the back of the room, away from the experimental setup, each time the researcher changed the glazing or shading throughout the whole experimental session. The researcher prompted the participant to imagine that this was the window view from their workstation and explained the definitions of each of the view aspects in the survey and answered any clarifying questions from the participant. After the diagnostic tests and participant survey practice were completed, researchers began the main experiment.

For the experiment, participants completed the visual acuity test, contrast sensitivity test, color matching, color arrangement, and satisfaction questions, in that order, for each of the 11 glazing and material cases. Each participant had a randomized material order.

## Measures

### Human visual performance tests

#### Visual acuity

**Figure 2:**
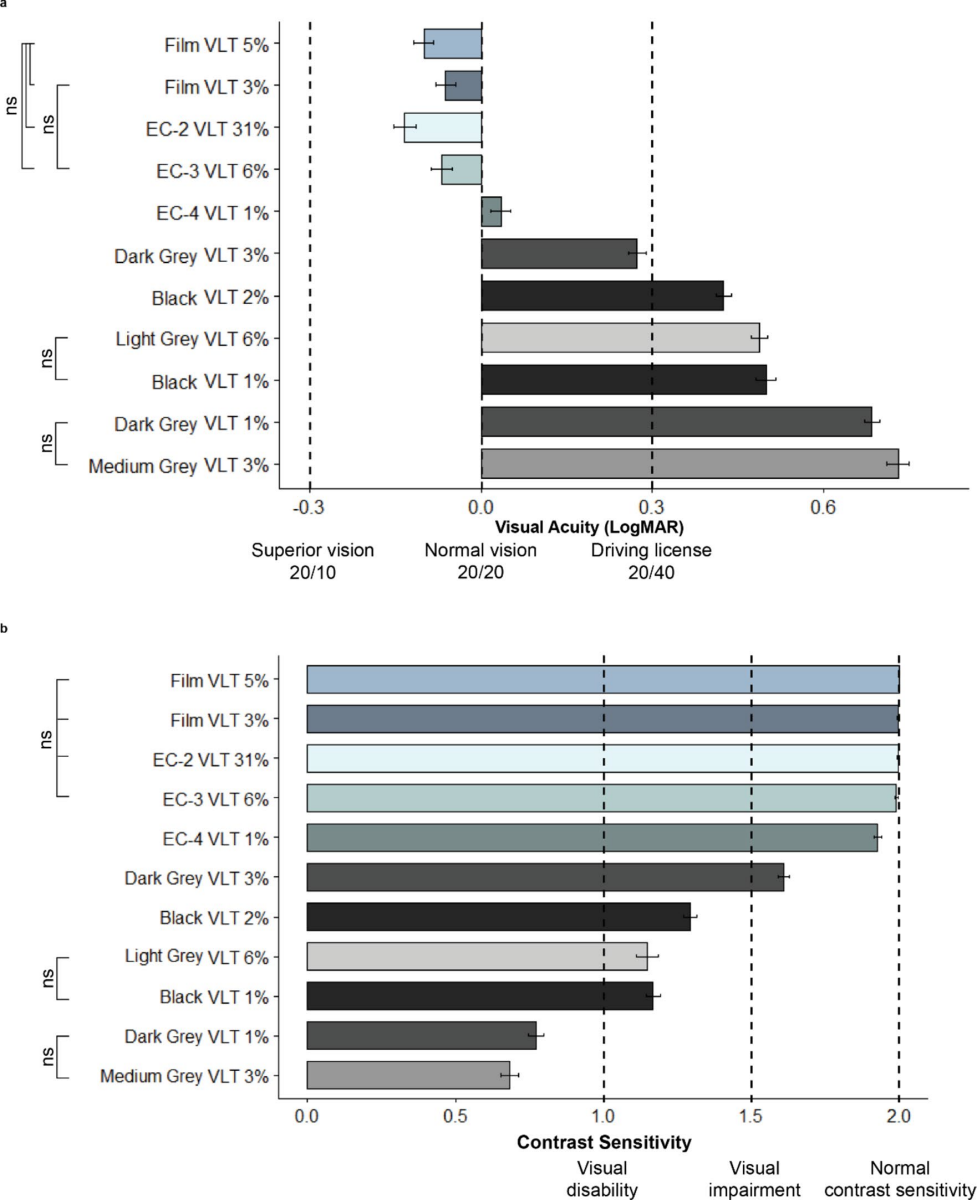
(**a**) Effect of window films, electrochromic (EC) glass, and fabric shade materials on visual acuity tested by a computer adaptation of the Snellen chart. Visual acuity represented by mean of LogMAR scores and standard error of the mean bars. ns annotations represent non-significant pairwise comparisons between materials (Supplementary Information online, Appendix D). Bar color represents material color. Film and EC glass versus fabric shade type yield better visual acuity performance; (**b**) Effect of window films, electrochromic (EC) glass, and fabric shade materials on contrast sensitivity tested by a computer adaptation of the Pelli-Robson chart. Contrast sensitivity represented by mean of sensitivity scores and standard error of the mean bars. ns annotations represent non-significant pairwise comparisons between materials (Supplementary Information online, Appendix D). Bar color represents material color. Film and EC glass versus fabric shade type yield better contrast sensitivity performance.

Visual acuity is a measure of the ability of the eye to see fine spatial details. It is typically tested by requiring a human participant to identify optotypes (numbers or letters) of progressively smaller sizes until they can no longer reliably identify the optotypes. The Snellen chart is an eye chart that can be used to measure visual acuity^30,31^ although there are more modern design of the charts. We adapted the chart test (Fig. 1c) to a digital format by presenting the optotypes that match the letter size in order to yield the corresponding visual angle on a computer screen considering the distance between the screen and participants^32^. The digital test projected one optotype at a time and if the participant read two out of three letters of the same size correctly, the size of the optotype was reduced and we repeated the same procedure until the participant failed to answer it correctly. The smallest optotype identified by the participant determines the participant’s acuity. The size of optotype ranges from 20/200 (legally blind) to 20/10 (superior vision) with 20/20 as a normal visual acuity level. A Snellen score of 20/20 indicates that an observer can resolve details as small as 1 min of visual angle at a distance of 20 feet. 20/200 vision means that a person can see details at 20 feet that people with normal vision can see at a distance of 200 feet. 20/10 vision means that a person can see details at 20 feet that people with normal vision can only see at a distance of 10 feet. In Fig. 2a, we visualize the visual acuity results using the LogMAR scale, with 0.0 corresponding to normal vision (20/20), 1.0 corresponding to legal blindness (20/200), and − 0.3 corresponding to superior vision (20/10). The LogMAR scale helps us to understand visual acuity scores more intuitively since 0.0 represents normal vision acuity, negative values are higher than normal vision, and positive values are lower than normal vision acuity.

#### Contrast sensitivity

Contrast sensitivity is the ability to detect subtle differences in luminance levels^33^. It is different from visual acuity, which measures the ability to recognize smaller and smaller letters as described above. Contrast sensitivity can be measured by using a contrast sensitivity chart such as the Pelli-Robson chart^32^. Letters on this chart have the same size, and the letters at the top of the chart are darkest and then gradually become lighter until they are almost impossible to recognize (Fig. 1d). Scoring is based on the ability to read the letters and subjects need to get at least two of the three letters correct before moving onto the next (lower) contrast level. Similar to the visual acuity test, we adapted the Pelli-Robson Chart to a digital format by projecting each digital letter, one at a time, to match the grayscale level of the original chart letter, unlike the original which shows all letters together^32^. The contrast sensitivity score ranges from 2.0 (normal contrast sensitivity), to 1.5 or less (visual impairment), to 1.0 or less (visual disability).

#### Color sensitivity

Optical properties of fenestration materials and chromaticity from (day)light can influence the subjective and physiological responses to a view^8,34,35^. Unnatural color appearance of view resulting from fenestration materials and lighting conditions can negatively affect occupant responses (e.g., satisfaction, emotion, comfort), which can impact the view perception of the occupant. There are a few studies that demonstrate the effect of colored light transmission through fenestration materials^36,37^ but there are not enough studies that demonstrate the effect of a shift in color perception within a view on occupant satisfaction. There are a few existing models for color quality assessment that align with human perception data^38^. Among the various color perception assessment methods, we adapted two tests: a color matching test and a color arrangement test.

### Color matching

Participants have 30 s to match each swatch of CIE Test Color Samples (TCS) 9–14^39^ using Google’s color picker web application (Fig. 1f and g^40^). TCS colors include TCS9 Strong Red, TCS10 Strong Yellow, TCS11 Strong Green, TCS12 Strong Blue, TCS13 Light Yellowish Pink, and TCS14 Moderate Olive Green. Participants complete the test by viewing both the TCS color swatch (on the left side of the monitor) and the color picker (on the right side of the monitor) through each of the 11 material cases. Researchers increased the computer cursor greatly in size so the participants could complete the test despite reduced view clarity. For result analysis, we used CAM16-UCS, a new color space addressing limitations of the current standard, CIE CAM02-UCS standard color space^41^. In CAM16-UCS, color is described by a′ (the amount of red–green), b′ (the amount of blue–yellow), and J′ (lightness) coordinates, ranging from 0 (black) to 100 (white). Color distance is calculated by the Euclidian distance (ΔE) between two points in this 3D color space. RGB to CAM16 color conversion inputs are available in Appendix E in Supplementary Information online.

### Color arrangement

We used the Color Challenge and Hue Test^42^, a simplified version of the Farnsworth Munsell 100 Hue Test that is offered in an online format (Fig. 1h). The hue test assesses the ability to isolate and arrange minute differences in various color targets. Participants are required to arrange 40 hues in 2 min and 30 s while having two anchors (red–yellow, yellow–green, green–blue, blue–red). A lower score is better with zero being a perfect score. The resulting circle graph (Fig. 1i) displays score by regions of the color spectrum, with points closer to the edge of the circle indicating lower hue discrimination.

### View satisfaction

Clarity is a primary variable of window view quality, and it may interact with other variables, such as content, because it can shift how occupants perceive the content, thereby affecting view satisfaction. To assess the effect of view clarity that resulted by different glazing and shade types on occupants’ view satisfaction, we measured view satisfaction using three scene images: a gray building view, a green pond view, and a street view with people (Fig. 1e). The scene images were carefully selected to represent a range of outdoor view content types from a previous study^43^. Participants rated seven view aspects of the scene images through the 11 different material cases from very dissatisfied to very satisfied on a 7-point Likert satisfaction scale (Supplementary Information online, Appendix C). We measured different facets of view satisfaction, including clarity of view, connection to the outside, visual privacy, color vividness, color naturalness, feeling of relaxation, and reflections/mirror effect.

### Statistical analysis

In the analysis of the results, we compared the mean values under different glazing and window shade conditions. While median values are more commonly applied to nominal or ordinal measurements, the mean provides more granular information (i.e., non-integer values) that describe the differences between groups of data.

The permutation test determined the statistical significance of the tested materials on human visual performance and view satisfaction within subjects. Permutation tests are non-parametric tests that solely rely on the assumption of exchangeability^44^. Among the different types of permutation tests, we used the General Symmetry Test using R package “coin” that accounts for the pair-wise comparison of the repeated measure within the same individual, and then assessed the difference between the conditions for each individual^45^. To increase the confidence of the discovered effects, we applied Bonferroni corrections to the *p* values. We assessed 55 pairwise comparisons between the tested materials, so a correction of *p* = 0.05/55 = 0.0009 was applied for significance.

We used the Linear Mixed Model (LMM) analysis to identify (1) the primary human visual performance factors to predict view satisfaction and (2) the predictive power of the optical properties of window and shade materials on human visual performance and view satisfaction. LMM is an extension of traditional linear models that allow both fixed and random effects, and it is a method for analyzing data that are non-independent, longitudinal or correlated^46^. LMM are valuable because they take part of the variability in the data within input (independent) variables that we do not need to have in the prediction model, like in our case the human subject. In other words, the variability connected to each subject is taken into account (random effect) but not included in the prediction model. Visual inspection of residual plots did not reveal any obvious deviations from homoscedasticity or normality, respecting the model assumptions of the LMM^47^. In order to assess the predicting power of each visual performance test result on satisfaction scores and the impact of each optical properties on view clarity, we evaluated each test result and each optical property as a sole fixed effect. We specified the participant ID as a random effect to control for personal variance. For the LMM analysis, we used R package “lmerTest”^44^ and another package “MuMIn” for the marginal R^2^ (the variance explained by the fixed effects) and conditional R^2^ (the total explanatory power of the model) calculations^48^. A R package, “mtcar” calculated the Variance Inflation Factor (VIF) to detect multicollinearity across the independent factors.

## Results

### Human visual performance

#### Visual acuity

Figure 2a shows the effect of glazing and window shade materials on visual acuity. Shade type (specular vs. fabric) generally influences visual acuity. Specular materials, except for EC-4 VLT 1%, achieve normal vision acuity (LogMAR 0.0; 20/20 Snellen score) even if their VLTs are reduced. In contrast, fabric shades lead to lower than normal vision acuity. With the exception of Dark Grey Fabric VLT 3% (the top bar of the fabric series), fabric shades led to acuity scores lower than LogMAR 0.3 (20/40). Under the same VLT (3%) and similar OF, the difference in color (Dark grey vs. Medium grey) between two fabric shades dropped visual acuity more than 50%, indicating the potential large effect of color difference alone in fabric shades.

The pair-wise comparisons between the Film VLT 5%, Film VLT 3%, EC-2 VLT 31% and EC-3 VLT 6%, and between Film VLT and EC-3 VLT 6%, are not statistically significant, indicating that their effects on participants’ visual acuity are very similar. By the same token, the pair-wise comparisons between Light Grey Fabric VLT 6% and Black Fabric VLT 1%, and between Dark Grey Fabric VLT 1% and Medium Grey Fabric VLT 3%, are not statistically significant, indicating that the interactions among OF, VLT, and color affect the final visual acuity scores. The rest of the pair-wise comparisons between the two materials are all statistically significant, indicating that the effect of each material on visual acuity is different.

#### Contrast sensitivity

Figure 2b represents the effect of glazing and window shade materials on contrast sensitivity. Specular materials yield normal contrast sensitivity performance except for EC-4 VLT 1%. In contrast, fabric shades lead to reduced contrast sensitivity (1.5 or lower) except for Dark Grey Fabric VLT 3%. Under the same VLT (3%) and similar OF, the difference in color (Dark grey vs. Medium grey) leads to a contrast sensitivity reduction of more than 50%. Unless the color is very dark, fabric shades with a lower OF (~ 5% below) yield a contrast sensitivity in the visual disability range (contrast sensitivity of 1.0 or lower).

The pair-wise comparisons between EC-2 VLT 31%, EC-2 VLT 31% and EC-3 VLT 6%, are not statistically significant, indicating that their effects on participants’ contrast sensitivity are very similar. By the same token, the pair-wise comparisons between Light Grey Fabric VLT 6% and Black Fabric VLT 1%, and between Dark Grey Fabric VLT 1% and Medium Grey Fabric VLT 3%, are not statistically significant, indicating the effect of color difference (Black vs. Light Grey) can be larger than the effect of the OF and VLT on contrast sensitivity.

#### Color sensitivity: color matching

**Figure 3:**
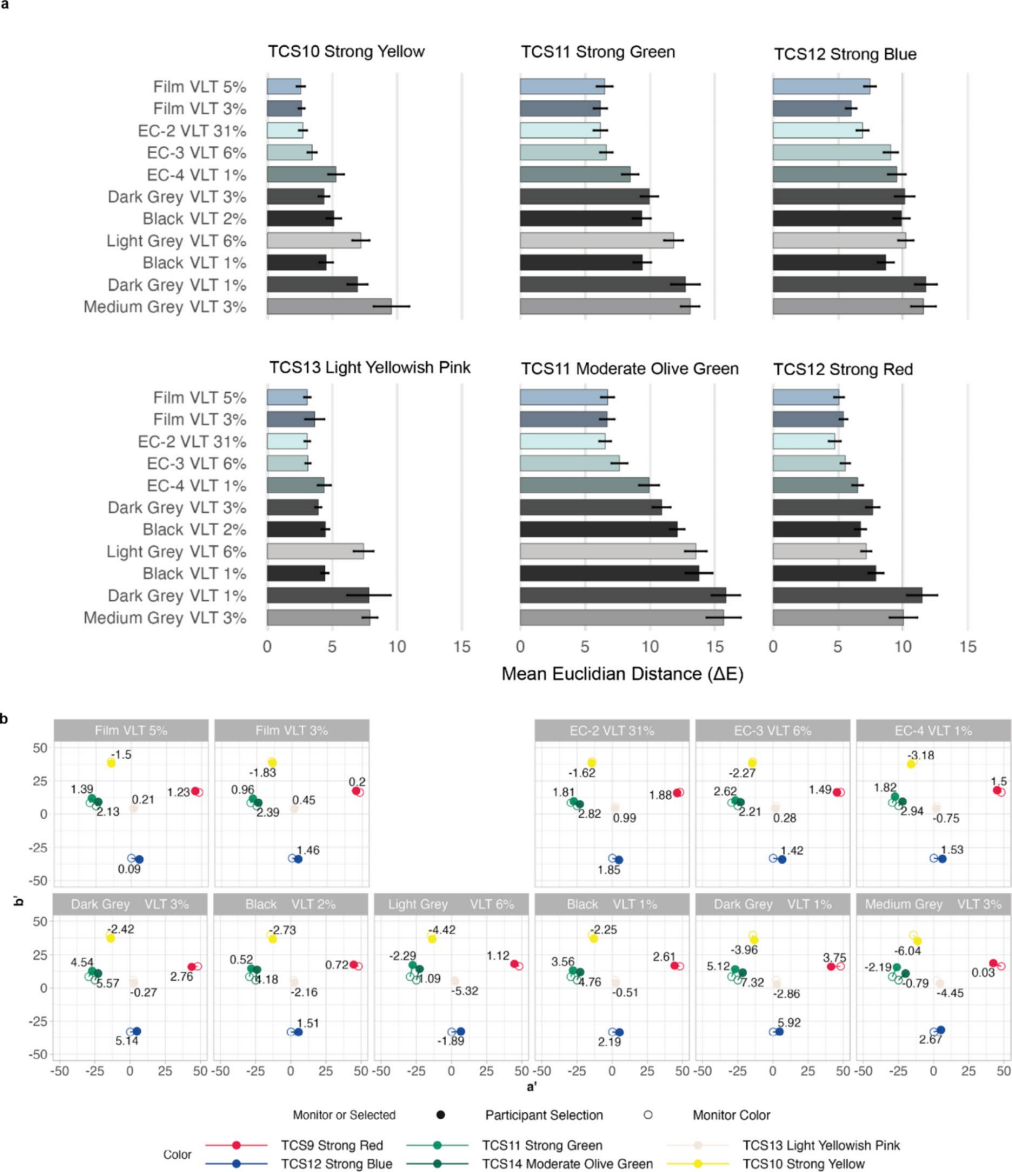
(**a**) Effect of window films, electrochromic glass, and fabric shades on participant ability to match TCS9-14 colors. Color matching accuracy represented by Euclidian distance (ΔE) in the CAM16 color space between actual color and participant selection. Mean of the scores and the standard error of the mean bars; (**b**) Effect of window films, electrochromic glass, and fabric shades on participant ability to match the hue of TCS9-14 colors. Hue shift is represented as the hue distance (Δa′b′) between TCS9-14 swatches and the mean participant color selection in CAM16 color space. Lightness shift (ΔJ′) is listed as a numeric label next to the corresponding points. The visualization method is adapted from^22^.

The ΔE results show that Strong Yellow and Light Yellowish Pink colors are most accurately matched, followed by Strong Red, and Strong Blue. Strong Green and Moderate Olive Green are least accurately matched (Fig. 3a). Fabric shades overall perform worse than specular cases.

We also assessed the perceived lightness (ΔJ′) differences. Participants’ TCS10 Strong Yellow selection is darker than the actual monitor color for all cases, meaning the color appears lighter. TCS13 Light Yellowish Pink has a minimal perceived lightness shift for higher acuity cases. In terms of openness factor, there is a slightly greater lightness shift for Black VLT 1% versus Black VLT 2% for the darker colors (Red, Greens, Blue). This suggests that a smaller openness factor may increase shade impact on lightness (J′).

If we only look at hue shift (Δa′b′), TCS12 Strong Blue appears slightly redder for every case, or in other words, closer to violet (Fig. 3b). TCS 11 and 14 green colors appear yellower.

#### Color sensitivity: color arrangement

**Figure 4:**
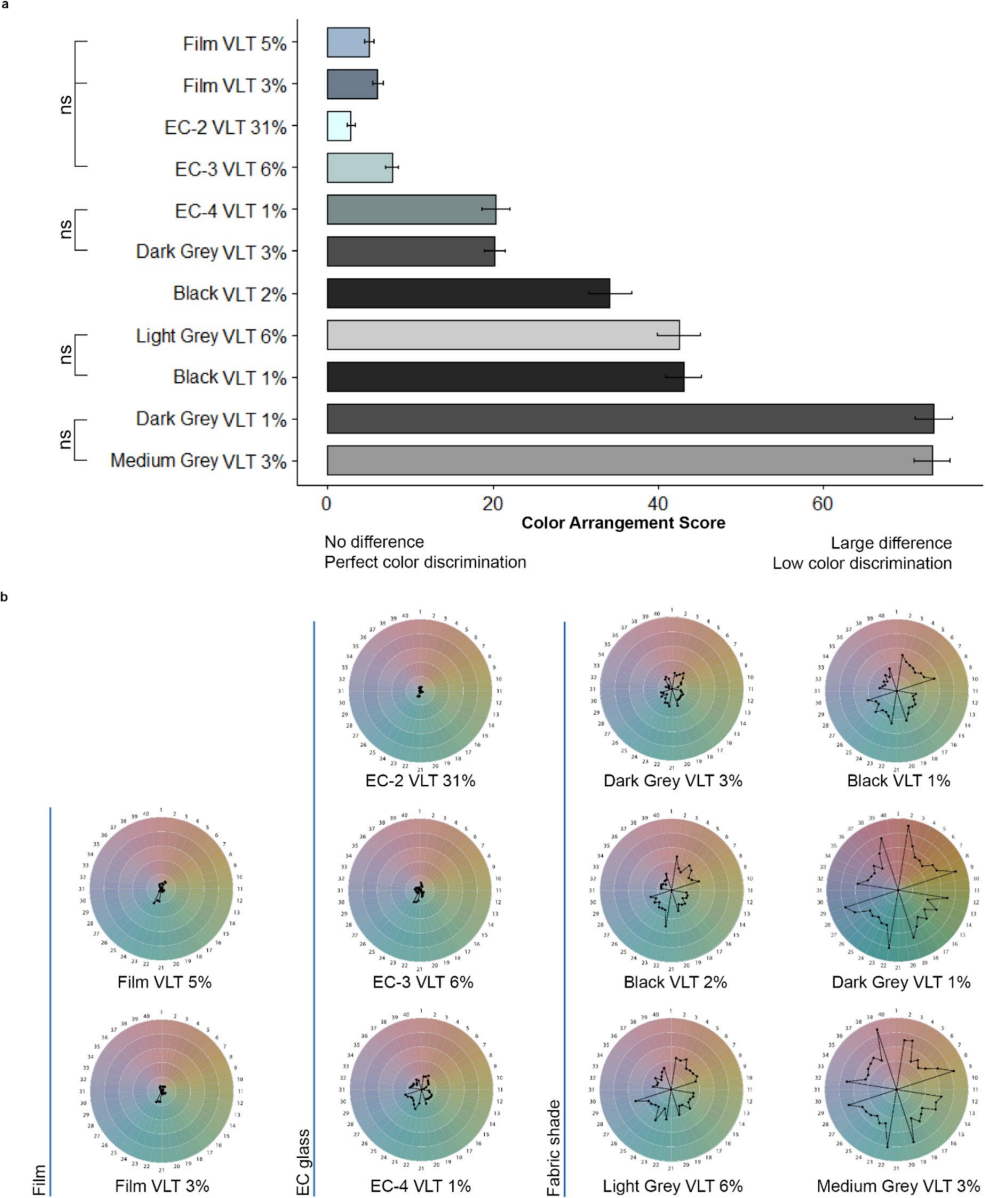
(**a**) Effect of glazing and window shade materials on the color arrangement test scores; Mean of the scores and the standard error of the mean bars. (**b**) Circle graph that displays the regions of the color spectrum where participants’ hue discrimination is low. Ns annotations represent non-significant pairwise comparisons between materials (Supplementary Information online, Appendix D). Bar color represents material color. Film and EC glass versus fabric shade type yield better color arrangement performance.

Figure 4a shows the effect of glazing and window shade materials on the color arrangement test scores. Specular materials perform better (i.e., better color discrimination) than fabric shades except for EC-4 VLT 1%. The performance for the majority of specular materials are not significantly different except for EC-4 VLT 1%. Interestingly, EC-4 VLT 1% and Dark Grey Fabric VLT 3% has equal performance, unlike visual acuity and contrast sensitivity, where the Dark Grey Fabric VLT 3% performs slightly worse. The pair-wise comparisons between the two films and EC-3 VLT 6% are equal, indicating their effects on participants’ color discrimination are very similar. By the same token, the pair-wise comparisons between Light Grey Fabric VLT 6% and Black Fabric VLT 1%, and between Dark Grey Fabric VLT 1% and Medium Grey Fabric VLT 3% are not statistically significantly different, indicating the interactions among OF, VLT, and color affect the participants’ ability in color discrimination. For the two fabric shades with the same OF and VLT (Fabric VLT 3%), the difference in color (Dark Grey vs. Medium Grey) reduces the color discrimination score by more than 70%.

Figure 4b shows the circle graph that displays score by regions of the color spectrum, with points closer to the edge of the circle indicating lower hue discrimination. Under both film conditions, participants’ ability to discriminate the blue–green hue was weaker than other colors. A similar trend was also found for EC-3 VLT 6% and EC-4 VLT 1%. There is a major shift with the fabric shades, with discrimination between blue–green and red–yellow worse than other colors. We found a relatively uniform effect on the color spectrum for fabric shades with the lowest visual acuity. These results are congruent with the fact that there is a correlation between visual acuity and color perception^49^.

### View satisfaction

**Figure 5:**
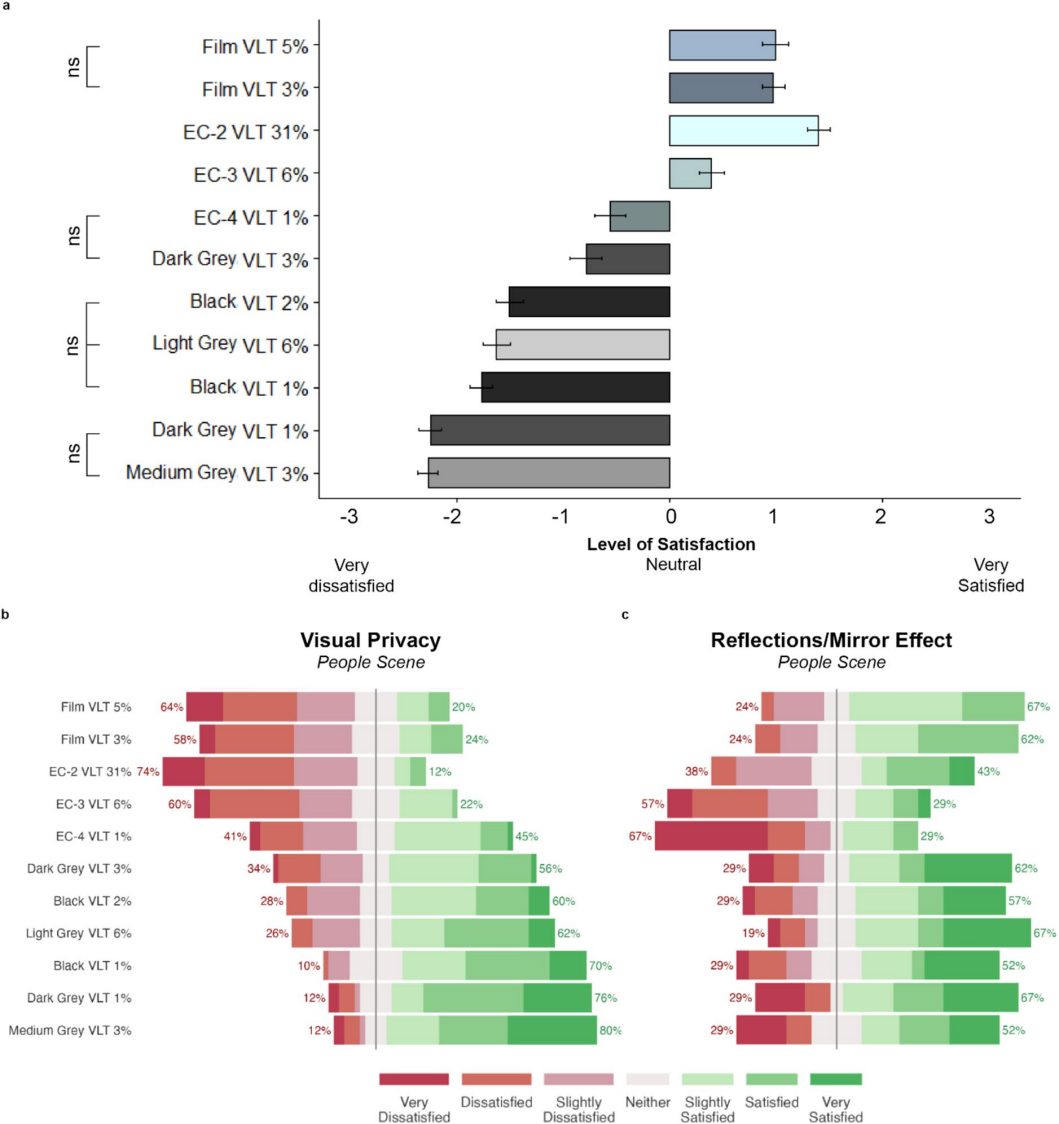
(**a**) Sum of satisfaction results for “clarity of view”, “connection to the outside”, “color vividness”, “color naturalness”, and “feeling of relaxation” averaged for Building, Pond, and People scenes. Mean of the scores and the standard error of the mean bars. ns annotations represent non-significant pairwise comparisons between materials Bar color represents material color (Supplementary Information online, Appendix D); (**b**) Satisfaction results for “visual privacy” for the street view with people scene (n = 50); (**c**) Satisfaction results for “reflections/mirror effect” for the street view with people scene (n = 21).

Satisfaction results for “clarity of view”, “connection to the outside”, “color vividness”, “color naturalness”, and “feeling of relaxation” follow a similar trend for each glazing and shading material trend, so the satisfaction metrics were combined in the next graphic representation (Fig. 5a). The general trend for these aspects is that people are most satisfied with the films, followed by electrochromic glass and least satisfied with fabric shades. One exception to this trend, EC-2 VLT 31% has a slightly better performance than the film tints. Of these five aspects, “feeling of relaxation” is most influenced by scene type (Supplementary Information online, Appendix F). Appendix G in Supplementary Information online presents more detailed patterns between the different satisfaction metrics and scene type.

Satisfaction results follow different trends for “visual privacy” and “reflections/mirror effect.” For “visual privacy,” lower clarity cases tend to have a more favorable rating (Fig. 5b). The street view with people scene yield greater dissatisfaction than the other two scenes except for the two lowest clarity cases (Medium Grey VLT 3% and Dark Grey VLT 1%). For “reflections/mirror effect,” EC-3 VLT 6% and EC-4 VLT 1% yield higher dissatisfaction ratings (Fig. 5c). For all other cases, median satisfaction scores are between neutral and satisfied.

### Effect of human visual performance on view satisfaction

The Linear Mixed Model (LMM) analysis shows that visual acuity and color matching (sum of six delta E values) scores were the two primary predictors for the sum of five view satisfaction results (Fig. 5a) and satisfaction with reflection effect. Table 2a shows the effect of human visual performance on view satisfaction prediction indicated by R^2^ values (average for the 3 scenes within each outcome variable). While the effects of contrast sensitivity and color arrangement scores on satisfaction scores are statistically significant, they showed multicollinearity with visual acuity scores. Hence, we only reported the LMM analysis results of the effect of the strongest predictor variables that are independent from each other in the table. The model could be described by the following Eq. (1):

1 $$ Y_{i} = a_{i} + \beta_{1} \cdot X_{1} + \beta_{2} \cdot X_{2} + \beta_{3} \cdot X_{3} + A_{i} + \varepsilon_{i} $$

**Table 2:** a. The effect of human visual performance on view satisfaction.

Outcome variable	Predictor variable	*a*	*β*	SE	*p* value	R_M_^2^	R_C_^2^
*a. Prediction models: the effect of human visual performance on view satisfaction*							
Sum of five view satisfaction results	intercept	− 0.79		0.10	< 0.001***	0.61	0.81
	Visual acuity		− 1.05	0.01	< 0.001***		
	Color matching		− 0.38	0.01	< 0.001***		
	Visual acuity: color matching		0.24	0.00	< 0.001***		
Visual privacy	intercept	0.71		0.08	< 0.001***	0.24	0.40
	Contrast sensitivity		− 0.70	0.02	< 0.001***		
	Color matching		0.06	0.02	0.003**		
	Contrast sensitivity: color matching		0.20	0.01	< 0.001***		
Reflection effect	intercept	0.34		0.20	< 0.001***	0.02	0.24
	Visual acuity		0.37	0.04	< 0.001***		
	Color matching		− 0.19	0.04	< 0.001***		
*b. Prediction models: the effect of the optical properties of fabric shades on human visual performance and view satisfaction*							
Visual acuity	intercept	0.54		0.01	< 0.001***	0.64	0.79
	VLT		− 0.13	0.13	< 0.001***		
	RS		0.06	0.00	< 0.001***		
Contrast sensitivity	intercept	1.10		0.02	< 0.001***	0.69	0.81
	VLT		0.25	0.00	< 0.001***		
	RS		− 0.13	0.00	< 0.001***		
Color matching	intercept	9.02		0.25	< 0.001***		
	VLT		− 1.03	0.03	< 0.001***	0.19	0.47
	RS		0.61	0.01	< 0.001***		
Color arrangement	intercept	52.12		1.64	< 0.001***	0.59	0.80
	VLT		− 16.48	0.07	< 0.001***		
	RS		7.88	0.14	< 0.001***		
Clarity of view satisfaction	intercept	− 2.23		0.11	< 0.001***	0.26	0.69
	VLT		0.51	0.00	< 0.001***		
	RS		− 0.24	0.00	< 0.001***		
Visual privacy	intercept	1.34		0.16	< 0.001***	0.02	0.68
	VLT		− 0.19	0.00	< 0.001**		
	RS		0.07	0.00	< 0.001***		
Reflection effect	intercept	0.51		0.42	< 0.001***	0.00	0.84
	VLT		0.10	0.02	< 0.001***		
	RS		− 0.03	0.00	< 0.001***		

The statistical information of the LMM models: estimated coefficient (β), standard error (SE), and statistical significance (*p* value), Marginal R2 (RM2), Conditional R2 (RC2). Predictor variables are normalized with standard scaling (i.e., the overall statistical summary of every variable has a mean value of zero and a unit variance value); b. The effect of the optical properties of fabric shades on human visual performance and view satisfaction (clarity of view and visual privacy). The statistical information of the LMM models: estimated coefficient (β), standard error (SE), and statistical significance (*p* value), Marginal R2 (RM2), Conditional R2 (RC2). Predictor variables ranges 0–100 (%).

The variables are defined as:

- ${Y}_{i}$: Occupant satisfaction with view (the sum of five view satisfaction or reflection effect)
- $${X}_{1}$$ : Visual acuity
- $${X}_{2}$$: Color matching
- ${X}_{3}$: Visual acuity: color matching (interaction)
- ${A}_{i}$: Random intercept due to subjects’ variation
- $${a}_{i}$$: Intercept
- $${\varepsilon }_{i}$$: Error term

For visual privacy satisfaction, contrast sensitivity and color matching scores were the two primary predictors. When predicting satisfaction with visual privacy and reflection effect, we found lower R^2^ values, indicating the effect of the predictor variables on the satisfaction scores are statistically significant but their practical utilities are quite low.

### View clarity and optical properties of fenestration materials

The optical properties of fenestration systems affect view clarity, influencing occupants' view perception. Based on the empirical findings, we developed LMM to evaluate the prediction power of the optical properties of fenestration materials on human visual performance and view satisfaction (clarity of view only, due to high correlation with other factors). When developing the prediction model with the entire dataset, we found that fenestration types (specular vs. fabric) significantly affected results, masking the influence of optical properties. Hence, we developed two separate models for each type. the specular material model showed the low prediction power of optical properties, possibly due to minimal or statistically insignificant effects on occupants. For brevity, we present the fabric shade model only in Table 2b. Appendix H in Supplementary Information online details the entire dataset and specular material model information.

#### Fabric shades

Table2-b shows the effect of the optical properties of fabric shades on the outcome variables. Among the optical properties provided by manufacturers, visible light transmittance (VLT) and solar reflectance (RS) are two primary predictors. Openness factor (OF) is another optical property that we considered throughout the experiment and a previous study^20^ identified as one of the variables in the View Clarity Index. However, when developing the LMM, we found that OF was highly correlated with VLT but had lower predictive power. To avoid multicollinearity and unreliable predictions, we dropped OF from the final models (i.e., VIF < 4;^50,51^. Konstantzos et al.^20^ used a parameter representing the fraction of direct-to-total light transmission (OF/VLT), which was also related to fabric reflectivity. However, our fabric shades results show that the OF/VLT parameter was not related to RS nor showed a statistical significance effect on the parameter. Hence, we included RS instead of the OF/VLT parameter. This incongruous finding may be due to the Konstanzos’ study including fabric shades with a wider range of OF/VLT (0.11 to 0.95) while the present study included a narrower OF/VLT ranges (0.67 to 0.90) or the different experimental settings (e.g., daylight conditions vs. controlled artificial lighting condition). The difference in the sample size (18 participants vs. 50 participants) could also be a potential reason for the disagreement. Even though there is some incongruence in the primary variables, the general trend found by the previous researcher holds: higher VLT and darker color (lower solar reflectance) generally correlate with higher visual performance and view satisfaction.

**Figure 6:**
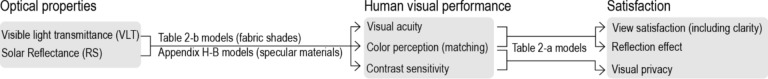
A flowchart illustrating the proposed framework for prediction models. Primary predictors for human visual performance and view satisfaction are shown in grey bubbles, with relevant Linear Mixed Models indicated by arrow lines. The predictive power of each model is detailed in Table 2 and Appendix H.

While VLT and RS had higher prediction power for human visual performance (ranging from 0.19 to 0.68), their prediction power for view satisfaction were lower (ranging from 0.00 to 0.26). This indicates that utilizing optical properties to predict the view satisfaction directly may not work since there are more factors influencing view satisfaction compared to human visual performance such as color, contrast, scene type (Supplementary Information online, Appendix F) and view size that are associated with the study settings. These limitations are discussed further in the following section. When using human visual performance as a predictor for view satisfaction, the prediction power is enhanced. Therefore, we recommend incorporating human visual performance tests to more accurately predict or inform view satisfaction in the analysis workflow. Figure 6 describes the proposed prediction framework that depicts the primary predictors for each LMM models and the workflow. The model could be described by the following Eq. (2):

2 $$ Y_{i} = a_{i} + \beta_{1} \cdot X_{1} + \beta_{2} \cdot X_{2} + \beta_{3} \cdot X_{3} + A_{i} + \varepsilon_{i} $$

The variables are defined as:

- $${Y}_{i}$$: Outcome variable
- $${X}_{n}:$$: Predictor variable
- ${A}_{i}$: Random intercept due to subjects’ variation
- $${a}_{i}$$: Intercept
- $${\varepsilon }_{i}$$: Error term

## Discussion

### Practical implications

The view clarity assessment workflow that we developed contributes to this field by evaluating multiple facets of view clarity in different shade materials, encompassing visual acuity, contrast sensitivity, color sensitivity, and view satisfaction. Our finding shows the significance of visual acuity and contrast sensitivity as key performance metrics in view clarity experiments. Visual acuity strongly predicts view satisfaction, while contrast sensitivity is the primary predictor for visual privacy. Among the color sensitivity tests, the color matching test is a better assessment method than the color arrangement test. While the color arrangement test gives an overview of how glazing and window shade materials affect different hue spectrums, the result is also highly correlated with visual acuity score, which can confound the results. In contrast, the color matching test is independent from visual acuity score. Hence, it can be one of the primary view clarity assessment methods along with visual acuity and contrast sensitivity. Among the six different CIE Test Color Samples the color matching test included, we found that 92% of results can be explained by four samples (i.e., Strong Blue, Red, Yellow and Light Yellowish Pink) using principal component analysis (PCA). Therefore, experimental time can be minimized by focusing on these four colors.

In the view satisfaction results, five satisfaction questions (clarity of view, connection to outside, color naturalness, color vividness, feeling of relaxation) showed similar trends. PCA analysis indicated that three questions (feeling of relaxation, color naturalness, clarity of view) covered 99.5% of the five satisfaction results. Visual privacy and reflection effects had different trends from the five satisfaction results, necessitating separate consideration when assessing how fenestration materials affects view satisfaction.

The findings from the present study with human subjects (evaluating how view clarity in fabric shades, window films, and EC glass affects occupants’ visual performance and view perception) benefit designers in selecting shade materials for fenestration systems. Key considerations include optical properties like VLT and RS, along with project-specific view content conditions (Supplementary Information online, Appendix F). In practice, designers select shade materials considering VLT to achieve desired daylight level and visual comfort. However, our study underscores that other factors such as shade type, RS and color of the shade materials as well as the primary design concern (e.g., view clarity, privacy, color naturalness) of the space would also need to be considered holistically. To maximize view clarity and contrast sensitivity, specular glazing materials are generally preferred, though those with very low VLT (e.g., 1%) may influence color sensitivity more than fabric shades. The VLT threshold for glare control under direct solar conditions varies with the sky conditions and sun positions across locations^24,52^. Therefore, careful consideration is required for spaces where conveying the natural view color amidst glare conditions is an important factor. When selecting a fabric shade, higher VLT and lower RS (darker color) would enhance human visual performance and view satisfaction, excluding visual privacy concerns.

### Study limitation

Conducting our study in a controlled lab space allowed us to control external variables, but it may not accurately reflect real-world conditions. First, the study room’s set-up with flood beam lighting fixtures, instead of sunlight, and intentional avoidance of glare for participant view clarity, presents limitations. Glare conditions, influenced by sun movement, glazing and shading, can affect visual comfort and perception metrics including acuity, contrast sensitivity, and color sensitivity^22^. Our focus was on developing an assessment workflow for glazing and shade systems’ view clarity, rather than testing real-world effects of the fenestration materials under different daylight conditions (e.g., intolerable glare and imperceptible glare). Second, we used a computer monitor to display view images but this may not have the same effect as a real outside view on satisfaction and other perceptual responses. The benefit, however, was that we could test multiple view content conditions under the controlled lighting environment, which would not have been possible in a study under real building conditions. Third, the lighting conditions created by the monitor screen and overhead lighting fixtures are lower than actual luminance levels one might experience in real buildings with windows to the outside. When checking the available climate data for a location close to the testing site, our illuminance of 10,300 lx is comparable to the low-end range of the hourly outdoor illuminance level. To take this into account, we tried to balance lighting ratios between exterior and interior by adjusting the diffused light fixture during lab setup. Nonetheless, this condition cannot represent all lighting conditions that one would experience in the perimeter area near real windows. The lower luminance levels in our study may impact participant color sensitivity. Perception of colorfulness (saturation) decreases with a decrease in luminance, known as the Hunt effect^53^. In addition, the color rendering on a display may not be the same as the real test and conditions. We calibrated the monitor using the built-in function of Apple iMac by selecting D65 as reference light source but the use of spectroradiometer would have created more accurate color testing conditions. This difference may have influenced participants’ color sensitivity. Finally, the small size of our window and monitor screen is also a limiting factor in our experiment. Though view access (i.e., the amount of the view an occupant can see from the viewing position) is not a primary focus of the current study, it is an important variable in view quality^13^. Our window sample is smaller than a typical office window, however the view access may be comparable to an occupant seated farther away from a larger window. As a result of our study limitations, we recommend conducting future studies on the impact of shading materials on view clarity in daylight conditions.

Due to experimental time constraints, we were limited to testing 11 material cases in the categories of window films, EC glass and roller shades. There is a wide range of shade materials and other window treatments in use today that also affect view clarity, such as frit glass, insect screens, and curtains. Further, our view clarity tests relied on a uniform material impact on the window view, excluding testing of binary shade materials such as venetian blinds (100% clarity or completely blocked), partially rolled-up fabric shades, shades with different interior- and exterior-facing colors, or materials with variable tint conditions by position on the window.

Lastly, we involved 50 participants; our sample size was larger than other studies (18 for^20^, 32 for^22^) but still quite limited. Moreover, most participants were college students. This may raise a question on the generalization of our finding to populations across different ages, cultures or countries, or for neurodiverse individuals. Some view satisfaction aspects, such as visual privacy, may have inherent cultural influences^54^, causing conflicting satisfaction scores for the same glazing and window shade conditions. Therefore, considering the potential moderating effect of cultural difference could be valuable in order to develop more reliable design standards and guidelines for view clarity.

Given the complexity of various fenestration systems and their associated visual parameters, more research in this field with a collaborative effort will allow us to collect and share data in order to develop more reliable and applicable prediction models.

## Conclusion

The present study assessed the effect of glazing and window shade materials (window films, electrochromic glass and fabric shades) on human visual performance and view satisfaction. To the best of our knowledge, this was the first study that compares the effects of specular and fabric shade materials on multiple facets of view clarity, including visual acuity, contrast sensitivity, color sensitivity, and view satisfaction, in a controlled environment. The use of artificial lighting fixtures and a computer screen to project visual tests and view images allowed us to test each material under a consistent lighting environment and simulated outdoor view condition, which is a common confounding factor for daylight research.

We concluded that shade type (specular vs. fabric) significantly influences human visual performance in most cases. Specular glazing materials achieved normal vision acuity and normal contrast sensitivity even if their VLT are reduced. The darkest EC tint level produced lower vision acuity and contrast sensitivity than other specular materials and was not statistically different from the best performing fabric shade for visual acuity and contrast sensitivity. For one pair of fabric shades with the same VLT (3%) and similar openness factor (OF) (5), the difference in color (Dark grey vs. Medium grey; 16% solar reflectance difference) reduced visual acuity and contrast sensitivity by more than 50% and color discrimination score by more than 70%. Color arrangement test results show that specular shades are worse for blue–green discrimination compared to other colors. For the fabric shades, except for the lowest visual acuity cases, participants’ ability in discriminating between green–blue and red–yellow colors is worse than discriminating between other colors.

The general trend for view satisfaction is that people are most satisfied with films, followed by EC glass, and least satisfied with fabric shades. We concluded that specular materials are favorable for most view aspects, but the darkest EC tint level presents a reflections challenge. Compared to specular materials, fabric shades are favorable for visual privacy, but they reduce satisfaction with other view aspects.

Based on LMM analyses, we found that visual acuity, contrast sensitivity and color matching tests are primary measures to evaluate the effect of glazing and window shade materials on occupant view satisfaction. VLT and RS values are primary optical properties in predicting human visual performance through the fabric shade materials. The models show the prediction accuracy ranges from 0.19 to 0.69, as determined by the marginal R^2^.

The findings from the present study contribute to continuing developments in the research-based view clarity assessment of fenestration systems. We propose an assessment workflow including the human visual performance aspects of visual acuity, contrast sensitivity, and color sensitivity, as well as corresponding view satisfaction. Additionally, the findings from this study’s view clarity assessment of window films, electrochromic glass, and fabric shades will help designers to select shade materials and advance their window design.

### Supplementary Information

Supplementary Information.

## Data Availability

Data and code available at the following locations: https://doi.org/10.5061/dryad.sn02v6xbc (forthcoming 2024) https://github.com/windowviewquality/view-clarity-experiment.
