# Supplementary material for: Assessing the impact of glazing and window shade systems on view clarity
Source: Sci Rep. 2024 Aug 8;14:18392. doi: 10.1038/s41598-024-69026-x (PMC11310397; doi:10.1038/s41598-024-69026-x)
Supplement: Supplementary file 1 — Supplementary Information. [file 41598_2024_69026_MOESM1_ESM.docx]

# Supplementary Information

## Appendix A

#### Experimental Set-up Lighting levels

|  | Luminance "P"* (cd/m^2) | Horizontal Illuminance (lux) at eye level | Vertical Illuminance (lux) at eye Level | Horizontal Illuminance (lux) desk level** | Luminance White Monitor (cd/m^2) | Luminance Black Monitor (cd/m^2) |
| --- | --- | --- | --- | --- | --- | --- |
| No Glass | 267.9 | 364.4 | 126 | 790.7 | 333.6 | 5.58 |
| Clear Glass | 122.2 | 361.4 | 98.2 | 544.6 | 184.3 | 5.97 |
| EC-2 VLT 31% | 38.22 | 357.7 | 64.6 | 301 | 32.01 | 3.58 |
| EC-3 VLT 6% | 13.4 | 357.1 | 59.4 | 253 | 12.33 | 1.55 |
| Light grey VLT 6% | 54.39 | 357.5 | 62.8 | 252 | 59.66 | 38.55 |
| Film VLT 3% | 9.04 | 357.1 | 59.4 | 245.5 | 12.82 | 0.73 |
| dark grey VLT 3% | 16.39 | 356.8 | 57 | 242.3 | 20.31 | 10.66 |
| Film VLT 5% | 15.67 | 357.4 | 60.8 | 254.9 | 19.24 | 2.82 |
| EC-4 VLT 1% | 3.06 | 356.8 | 57.6 | 233.7 | 3.72 | 0.55 |
| black VLT 1% | 8.1 | 356.7 | 55.4 | 231.2 | 9.25 | 5.85 |
| black VLT 2% | 9.8 | 356.7 | 55.8 | 234.1 | 12.26 | 6.17 |
| dark grey VLT 1% | 6.07 | 356.5 | 55.4 | 229.1 | 6.53 | 5.09 |
| medium grey VLT 3% | 30.92 | 357.2 | 59.2 | 238.1 | 28.65 | 24.11 |

* Taken with acuity largest letter P, measuring circle half on bottom P line and half on white space in the middle of P

** Desk level (30" above floor, 34" back from midpoint of glass - in front of participant)

Luminance values measured with Konica Minolta Luminance Meter LS-160, illuminance values measured with LI-COR Light Meter LI-250

## Appendix B

#### Material Product Information by VLT Ranges

| VLT Range | Name | Manufacturer | Product Name | Description |
| --- | --- | --- | --- | --- |
| VLT 31% | EC-2 VLT 31% | View | View Gen 4 IGU | Tint level 2 |
| VLT 5-6% | EC-3 VLT 6% | View | View Gen 4 IGU | Tint level 3 |
|  | Light Grey VLT 6% | MechoShade | 2113 Grey (ThermoVeil) | Fabric |
|  | Film VLT 5% | 3M | CS05 | Film |
| VLT 2-3% | Film VLT 3% | 3M | NA05 | Film |
|  | Dark Grey VLT 3% | MechoShade | 1922 Mocha (SoHo) | Fabric |
|  | Medium Grey VLT 3% | MechoShade | 1363 Grey (EcoVeil) | Fabric |
|  | Black VLT 2% | MechoShade | 1918 Black Brown (SoHo) | Fabric |
| VLT 1% | EC-4 VLT 1% | View | View Gen 4 IGU | Tint level 4 |
|  | Black VLT 1% | MechoShade | 1618 Black Brown (SoHo) | Fabric |
|  | Dark Grey VLT 1% | MechoShade | 1112 Charcoal (SoHo) | Fabric |

#### No glass and clear glass conditions

In addition to the eleven glazing and shade materials, we tested no glass and clear glass (SunGuard SN54, Guardian Glass) conditions as baseline conditions. No glass cases were presented first as practice rounds for human visual performance tests. The clear glass case was included within the random order of 11 glazing and shade materials for both human visual performance tests and view satisfaction, but only with the last 15 participants. We found that visual acuity, contrast sensitivity and color arrangement test did not show a learning effect while color matching results showed a learning effect (i.e., the scores under no glass condition were worse than those under clear glass). This indicates that it is essential to have practice rounds for the color matching test to prevent the learning effect. Below, we summarize the results (mean values) under no glass and clear glass conditions.

| Results | No glass (n=50) | Clear glass (n=15) |
| --- | --- | --- |
| Visual acuity | -0.19 | -0.22 |
| Contrast sensitivity | 2 | 2 |
| Color sensitivity: color matching | 1.64 | 2.40 |
| Color sensitivity: color arrangement | 6.20 | 4.84 |
| Sum of five view satisfaction | NA | 1.90 |
| Visual privacy satisfaction | NA | -0.18 |
| Reflection effect satisfaction | NA | 1.31 |

## Appendix C

#### Satisfaction Survey Questions


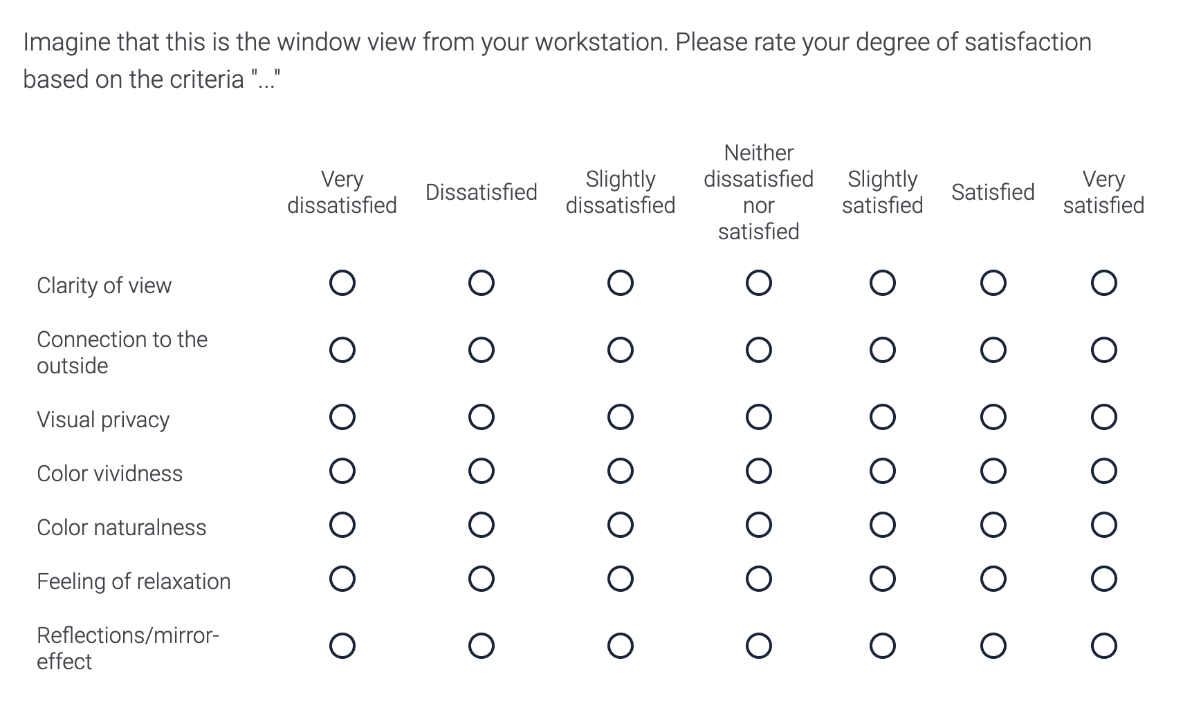


## Appendix D

#### Pair-wise comparison results: View acuity

Z-statistics, statistical significance (p-value; permutation test, Bonferroni corrected significance level: 0.05/55 = 0.0009*; 0.01/55 = 0.0002**; 0.001/55= 0.00002***)

| Case 1 | Case 2 | *Z* | *p-value* |
| --- | --- | --- | --- |
| Film VLT 5% | Film VLT 3% | 2.43 | 0.02 ns |
|  | EC-2 VLT 31% | 1.98 | 0.05 ns |
|  | EC-3 VLT 6% | 1.91 | 0.06 ns |
|  | EC-4 VLT 1% | 5.24 | < 2.0e-05 *** |
|  | Dark Grey VLT 3% | 6.69 | < 2.0e-05 *** |
|  | Medium Grey VLT 3% | 6.88 | < 2.0e-05 *** |
|  | Black VLT 2% | 6.85 | < 2.0e-05 *** |
|  | Light Grey VLT 6% | 6.89 | < 2.0e-05 *** |
|  | Black VLT 1% | 6.93 | < 2.0e-05 *** |
|  | Dark Grey VLT 1% | 6.95 | < 2.0e-05 *** |
| Film VLT 3% | EC-2 VLT 31% | 3.63 | 0.0002 ** |
|  | EC-3 VLT 6% | 1.91 | 0.06 ns |
|  | EC-4 VLT 1% | 4.71 | < 2.0e-05 *** |
|  | Dark Grey VLT 3% | 6.85 | < 2.0e-05 *** |
|  | Medium Grey VLT 3% | 6.85 | < 2.0e-05 *** |
|  | Black VLT 2% | 6.85 | < 2.0e-05 *** |
|  | Light Grey VLT 6% | 6.87 | < 2.0e-05 *** |
|  | Black VLT 1% | 6.85 | < 2.0e-05 *** |
|  | Dark Grey VLT 1% | 6.94 | < 2.0e-05 *** |
| EC-2 VLT 31% | EC-3 VLT 6% | 3.67 | 0.0002 ** |
|  | EC-4 VLT 1% | 5.98 | < 2.0e-05 *** |
|  | Dark Grey VLT 3% | 6.77 | < 2.0e-05 *** |
|  | Medium Grey VLT 3% | 6.89 | < 2.0e-05 *** |
|  | Black VLT 2% | 6.89 | < 2.0e-05 *** |
|  | Light Grey VLT 6% | 6.88 | < 2.0e-05 *** |
|  | Black VLT 1% | 6.88 | < 2.0e-05 *** |
|  | Dark Grey VLT 1% | 6.97 | < 2.0e-05 *** |
| EC-3 VLT 6% | EC-4 VLT 1% | 4.82 | < 2.0e-05 *** |
|  | Dark Grey VLT 3% | 6.56 | < 2.0e-05 *** |
|  | Medium Grey VLT 3% | 6.86 | < 2.0e-05 *** |
|  | Black VLT 2% | 6.85 | < 2.0e-05 *** |
|  | Light Grey VLT 6% | 6.85 | < 2.0e-05 *** |
|  | Black VLT 1% | 6.86 | < 2.0e-05 *** |
|  | Dark Grey VLT 1% | 6.95 | < 2.0e-05 *** |
| EC-4 VLT 1% | Dark Grey VLT 3% | 6.17 | < 2.0e-05 *** |
|  | Medium Grey VLT 3% | 6.82 | < 2.0e-05 *** |
|  | Black VLT 2% | 6.70 | < 2.0e-05 *** |
|  | Light Grey VLT 6% | 6.77 | < 2.0e-05 *** |
|  | Black VLT 1% | 6.74 | < 2.0e-05 *** |
|  | Dark Grey VLT 1% | 6.90 | < 2.0e-05 *** |
|  |  |  |  |
| Dark Grey VLT 3% | Medium Grey VLT 3% | 6.69 | < 2.0e-05 *** |
|  | Black VLT 2% | 6.00 | < 2.0e-05 *** |
|  | Light Grey VLT 6% | 6.32 | < 2.0e-05 *** |
|  | Black VLT 1% | 6.32 | < 2.0e-05 *** |
|  | Dark Grey VLT 1% | 6.89 | < 2.0e-05 *** |
| Medium Grey VLT 3% | Black VLT 2% | 6.42 | < 2.0e-05 *** |
|  | Light Grey VLT 6% | 6.11 | < 2.0e-05 *** |
|  | Black VLT 1% | 5.76 | < 2.0e-05 *** |
|  | Dark Grey VLT 1% | 1.85 | 0.06 ns |
| Black VLT 2% | Light Grey VLT 6% | 3.76 | < 2.0e-05 *** |
|  | Black VLT 1% | 4.25 | < 2.0e-05 *** |
|  | Dark Grey VLT 1% | 6.59 | < 2.0e-05 *** |
| Light Grey VLT 6% | Black VLT 1% | 0.81 | 0.42 ns |
|  | Dark Grey VLT 1% | 6.12 | < 2.0e-05 *** |
| Black VLT 1% | Dark Grey VLT 1% | 5.81 | < 2.0e-05 *** |

#### Pair-wise comparison results: Contrast sensitivity

Z-statistics, statistical significance (p-value; permutation test, Bonferroni corrected significance level: 0.05/55 = 0.0009*; 0.01/55 = 0.0002**; 0.001/55= 2.0e-05***)

| Case 1 | Case 2 | *Z* | *p-value* |
| --- | --- | --- | --- |
| EC-2 VLT 31% | EC-3 VLT 6% | 1 | 0.32 ns |
|  | Light Grey VLT 6% | 6.7 | < 2.0e-05 *** |
|  | Film VLT 5% | 1 | 0.32 ns |
|  | Dark Grey VLT 3% | 6.64 | < 2.0e-05 *** |
|  | Film VLT 3% | 0 | 1 ns |
|  | EC-4 VLT 1% | 4.27 | 1.9e-05 ** |
|  | Black VLT 1% | 6.92 | < 2.0e-05 *** |
|  | Black VLT 2% | 6.90 | < 2.0e-05 *** |
|  | Dark Grey VLT 1% | 7.00 | < 2.0e-05 *** |
|  | Medium Grey VLT 3% | 6.98 | < 2.0e-05 *** |
| EC-3 VLT 6% | Light Grey VLT 6% | 6.77 | < 2.0e-05 *** |
|  | Film VLT 5% | 1.73 | 0.08 ns |
|  | Dark Grey VLT 3% | 6.64 | < 2.0e-05 *** |
|  | Film VLT 3% | 1 | 0.32 ns |
|  | EC-4 VLT 1% | 3.90 | 9.6e-05** |
|  | Black VLT 1% | 6.90 | < 2.0e-05 *** |
|  | Black VLT 2% | 6.88 | < 2.0e-05 *** |
|  | Dark Grey VLT 1% | 6.99 | < 2.0e-05 *** |
|  | Medium Grey VLT 3% | 6.97 | < 2.0e-05 *** |
| Light Grey VLT 6% | Film VLT 5% | 6.77 | < 2.0e-05 *** |
|  | Dark Grey VLT 3% | 6.37 | < 2.0e-05 *** |
|  | Film VLT 3% | 6.77 | < 2.0e-05 *** |
|  | EC-4 VLT 1% | 6.73 | < 2.0e-05 *** |
|  | Black VLT 1% | 0.58 | 0.56 ns |
|  | Black VLT 2% | 3.37 | 0.0007 * |
|  | Dark Grey VLT 1% | 5.70 | < 2.0e-05 *** |
|  | Medium Grey VLT 3% | 6.02 | < 2.0e-05 *** |
| Film VLT 5% | Dark Grey VLT 3% | 6.65 | < 2.0e-05 *** |
|  | Film VLT 3% | 1 | 0.32 ns |
|  | EC-4 VLT 1% | 4.38 | < 2.0e-05 *** |
|  | Black VLT 1% | 6.91 | < 2.0e-05 *** |
|  | Black VLT 2% | 6.90 | < 2.0e-05 *** |
|  | Dark Grey VLT 1% | 6.99 | < 2.0e-05 *** |
|  | Medium Grey VLT 3% | 6.98 | < 2.0e-05 *** |
| Dark Grey 5 VLT 3% | Film VLT 3% | 6.64 | < 2.0e-05 *** |
|  | EC-4 VLT 1% | 6.33 | < 2.0e-05 *** |
|  | Black VLT 1% | 6.69 | < 2.0e-05 *** |
|  | Black VLT 2% | 6.33 | < 2.0e-05 *** |
|  | Dark Grey VLT 1% | 6.89 | < 2.0e-05 *** |
|  | Medium Grey VLT 3% | 6.85 | < 2.0e-05 *** |
| Film VLT 3% | EC-4 VLT 1% | 4.27 | < 2.0e-05 *** |
|  | Black VLT 1% | 6.91 | < 2.0e-05 *** |
|  | Black VLT 2% | 6.89 | < 2.0e-05 *** |
|  | Dark Grey VLT 1% | 6.99 | < 2.0e-05 *** |
|  | Medium Grey VLT 3% | 6.98 | < 2.0e-05 *** |
| EC-4 VLT 1% | Black VLT 1% | 6.89 | < 2.0e-05 *** |
|  | Black VLT 2% | 6.84 | < 2.0e-05 *** |
|  | Dark Grey VLT 1% | 6.99 | < 2.0e-05 *** |
|  | Medium Grey VLT 3% | 6.95 | < 2.0e-05 *** |
| Black VLT 1% | Black VLT 2% | 4.24 | < 2.0e-05 *** |
|  | Dark Grey VLT 1% | 6.31 | < 2.0e-05 *** |
|  | Medium Grey VLT 3% | 6.36 | < 2.0e-05 *** |
| Black VLT 2% | Dark Grey VLT 1% | 6.63 | < 2.0e-05 *** |
|  | Medium Grey VLT 3% | 6.67 | < 2.0e-05 *** |
| Dark Grey 1 VLT 1% | Medium Grey VLT 3% | 2.43 | 0.01 ns |

#### Pair-wise comparison results: Color arrangement

Z-statistics, statistical significance (p-value; permutation test, Bonferroni corrected significance level: 0.05/55 = 0.0009*; 0.01/55 = 0.0002**; 0.001/66= 2.0e-05***)

| Case 1 | Case 2 | *Z* | *p-value* |
| --- | --- | --- | --- |
| EC-2 VLT 31% | EC-3 VLT 6% | 5.26 | < 2.0e-05 *** |
|  | Light Grey VLT 6% | 6.49 | < 2.0e-05 *** |
|  | Film VLT 5% | 3.98 | 6.86e-05 ** |
|  | Dark Grey VLT 3% | 6.38 | < 2.0e-05 *** |
|  | Film VLT 3% | 4.17 | 3.02e-05 ** |
|  | EC-4 VLT 1% | 6.09 | < 2.0e-05 *** |
|  | Black VLT 1% | 6.66 | < 2.0e-05 *** |
|  | Black VLT 2% | 6.22 | < 2.0e-05 *** |
|  | Dark Grey VLT 1% | 6.93 | < 2.0e-05 *** |
|  | Medium Grey VLT 3% | 6.93 | < 2.0e-05 *** |
| EC-3 VLT 6% | Light Grey VLT 6% | 6.93 | < 2.0e-05 *** |
|  | Film VLT 5% | 3.30 | 0.001 ns |
|  | Dark Grey VLT 3% | 6.38 | < 2.0e-05 *** |
|  | Film VLT 3% | 2.41 | 0.02 ns |
|  | EC-4 VLT 1% | 5.78 | < 2.0e-05 *** |
|  | Black VLT 1% | 6.50 | < 2.0e-05 *** |
|  | Black VLT 2% | 5.94 | < 2.0e-05 *** |
|  | Dark Grey VLT 1% | 6.90 | < 2.0e-05 *** |
|  | Medium Grey VLT 3% | 6.90 | < 2.0e-05 *** |
| Light Grey VLT 6% | Film VLT 5% | 6.41 | < 2.0e-05 *** |
|  | Dark Grey VLT 3% | 6.07 | < 2.0e-05 *** |
|  | Film VLT 3% | 6.39 | < 2.0e-05 *** |
|  | EC-4 VLT 1% | 5.48 | < 2.0e-05 *** |
|  | Black VLT 1% | 0.27 | 0.79 ns |
|  | Black VLT 2% | 3.49 | 0.0005 * |
|  | Dark Grey VLT 1% | 6.01 | < 2.0e-05 *** |
|  | Medium Grey VLT 3% | 6.26 | < 2.0e-05 *** |
| Film VLT 5% | Dark Grey VLT 3% | 6.10 | < 2.0e-05 *** |
|  | Film VLT 3% | 1.32 | 0.19 ns |
|  | EC-4 VLT 1% | 5.86 | < 2.0e-05 *** |
|  | Black VLT 1% | 6.60 | < 2.0e-05 *** |
|  | Black VLT 2% | 6.06 | < 2.0e-05 *** |
|  | Dark Grey VLT 1% | 6.91 | < 2.0e-05 *** |
|  | Medium Grey VLT 3% | 6.91 | < 2.0e-05 *** |
| Dark Grey VLT 3% | Film VLT 3% | 6.07 | < 2.0e-05 *** |
|  | EC-4 VLT 1% | 0.09 | 0.92 ns |
|  | Black VLT 1% | 6.20 | < 2.0e-05 *** |
|  | Black VLT 2% | 5.05 | < 2.0e-05 *** |
|  | Dark Grey VLT 1% | 6.77 | < 2.0e-05 *** |
|  | Medium Grey VLT 3% | 6.87 | < 2.0e-05 *** |
| Film VLT 3% | EC-4 VLT 1% | 6.87 | < 2.0e-05 *** |
|  | Black VLT 1% | 6.61 | < 2.0e-05 *** |
|  | Black VLT 2% | 6.04 | < 2.0e-05 *** |
|  | Dark Grey VLT 1% | 6.90 | < 2.0e-05 *** |
|  | Medium Grey VLT 3% | 6.92 | < 2.0e-05 *** |
| EC-4 VLT 1% | Black VLT 1% | 5.91 | < 2.0e-05 *** |
|  | Black VLT 2% | 4.44 | < 2.0e-05 *** |
|  | Dark Grey VLT 1% | 6.77 | < 2.0e-05 *** |
|  | Medium Grey VLT 3% | 6.75 | < 2.0e-05 *** |
| Black VLT 1% | Black VLT 2% | 3.49 | 0.0005 * |
|  | Dark Grey VLT 1% | 6.04 | < 2.0e-05 *** |
|  | Medium Grey VLT 3% | 6.49 | < 2.0e-05 *** |
| Black VLT 2% | Dark Grey VLT 1% | 6.11 | < 2.0e-05 *** |
|  | Medium Grey VLT 3% | 6.44 | < 2.0e-05 *** |
| Dark Grey 1 VLT 1% | Medium Grey VLT 3% | 0.08 | 0.94 ns |

#### Pair-wise comparison results: Satisfaction sum 5 dimensions combined avg of 3 scenes

Z-statistics, statistical significance (p-value; permutation test, Bonferroni corrected significance level: 0.05/55 = 0.0009*; 0.01/55 = 0.0002**; 0.001/55= 2.0e-05***)

| Case 1 | Case 2 | *Z* | *p-value* |
| --- | --- | --- | --- |
| EC-2 VLT 31% | EC-3 VLT 6% | -5.5378 | < 2.0e-05 *** |
|  | Light Grey VLT 6% | -6.6734 | < 2.0e-05 *** |
|  | Film VLT 5% | 3.9525 | 7.733e-05 **  People ns |
|  | Dark Grey VLT 3% | -6.3372 | < 2.0e-05 *** |
|  | Film VLT 3% | 3.723 | 0.0001969 *  People ns |
|  | EC-4 VLT 1% | -6.0274 | < 2.0e-05 *** |
|  | Black VLT 1% | -6.7634 | < 2.0e-05 *** |
|  | Black VLT 2% | -6.7133 | < 2.0e-05 *** |
|  | Dark Grey VLT 1% | -6.8318 | < 2.0e-05 *** |
|  | Medium Grey VLT 3% | -6.8392 | < 2.0e-05 *** |
| EC-3 VLT 6% | Light Grey VLT 6% | -6.4887 | < 2.0e-05 *** |
|  | Film VLT 5% | -4.1718 | 3.022e-05 **  People ns |
|  | Dark Grey VLT 3% | -5.7654 | < 2.0e-05 *** |
|  | Film VLT 3% | -4.4714 | < 2.0e-05 *** |
|  | EC-4 VLT 1% | -5.4189 | < 2.0e-05 *** |
|  | Black VLT 1% | -6.6188 | < 2.0e-05 *** |
|  | Black VLT 2% | -6.4821 | < 2.0e-05 *** |
|  | Dark Grey VLT 1% | -6.6511 | < 2.0e-05 *** |
|  | Medium Grey VLT 3% | -6.6646 | < 2.0e-05 *** |
| Light Grey VLT 6% | Film VLT 5% | -6.5259 | < 2.0e-05 *** |
|  | Dark Grey 5 VLT 3% | -5.0457 | < 2.0e-05 *** |
|  | Film VLT 3% | -6.6164 | < 2.0e-05 *** |
|  | EC-4 VLT 1% | -5.6052 | < 2.0e-05 *** |
|  | Black VLT 1% | 1.5079 | 0.1316 ns |
|  | Black VLT 2% | -1.249 | 0.2117 ns |
|  | Dark Grey VLT 1% | -4.9536 | < 2.0e-05 ***  Building ns |
|  | Medium Grey VLT 3% | -5.3776 | < 2.0e-05 ***  Building ns |
| Film VLT 5% | Dark Grey VLT 3% | -5.9814 | < 2.0e-05 *** |
|  | Film VLT 3% | 0.48748 | 0.6259 ns |
|  | EC-4 VLT 1% | -5.6013 | < 2.0e-05 *** |
|  | Black VLT 1% | -6.6678 | < 2.0e-05 *** |
|  | Black VLT 2% | -6.5569 | < 2.0e-05 *** |
|  | Dark Grey VLT 1% | -6.7142 | < 2.0e-05 *** |
|  | Medium Grey VLT 3% | -6.7367 | < 2.0e-05 *** |
| Dark Grey VLT 3% | Film VLT 3% | -6.2223 | < 2.0e-05 *** |
|  | EC-4 VLT 1% | -1.9248 | 0.05426 ns |
|  | Black VLT 1% | -5.7419 | < 2.0e-05 *** |
|  | Black VLT 2% | -5.4415 | < 2.0e-05 *** |
|  | Dark Grey VLT 1% | -5.9022 | < 2.0e-05 *** |
|  | Medium Grey VLT 3% | -5.9024 | < 2.0e-05 *** |
| Film VLT 3% | EC-4 VLT 1% | -5.9366 | < 2.0e-05 *** |
|  | Black VLT 1% | -6.7709 | < 2.0e-05 *** |
|  | Black VLT 2% | -6.6588 | < 2.0e-05 *** |
|  | Dark Grey VLT 1% | -6.7813 | < 2.0e-05 *** |
|  | Medium Grey VLT 3% | -6.7619 | < 2.0e-05 *** |
| EC-4 VLT 1% | Black VLT 1% | -5.9682 | < 2.0e-05 *** |
|  | Black VLT 2% | -5.1638 | < 2.0e-05 *** |
|  | Dark Grey VLT 1% | -6.0903 | < 2.0e-05 *** |
|  | Medium Grey VLT 3% | -6.1172 | < 2.0e-05 *** |
| Black VLT 1% | Black VLT 2% | -2.6641 | 0.007719 ns |
|  | Dark Grey VLT 1% | -4.8667 | < 2.0e-05 ***  Building ns |
|  | Medium Grey VLT 3% | -4.5175 | < 2.0e-05 ***  Building ns |
| Black VLT 2% | Dark Grey VLT 1% | -4.805 | < 2.0e-05 ***  Building ns |
|  | Medium Grey VLT 3% | -5.0015 | < 2.0e-05 ***  Building ns |
| Dark Grey VLT 1% | Medium Grey VLT 3% | 0.29089 | 0.7711 ns |

## Appendix E

#### CAM16 color Space and chromatic adaptation transformation

D65 reference white: *X*_w_ = 95.047, *Y*_w_ = 100, *Z*_w_ = 108.883

CIECAM02 Standard Inputs:

Background luminance *Y*_b_ = 20 cd/m^2^, Surround parameter *F* = 1, Surround parameter *N*_c_ = 1, Surround parameter *c* = 0.69, Luminance of adapting field *L*_A_ = 100 cd/m^2^,

Degree of adaptation *D* = 1, Reference illuminant *Y*_w_ = 100,

*k* = 0.0020, *F*_L_ = 0.7937, *n* = 0.2, *N*_bb_ = *N*_cb_ = 1.0003, *z* = 1.9272

## Appendix F

#### Effect of scene type on view satisfaction

The table below shows the effect of visual acuity scores and scene type on the view satisfaction, ranked from highest to lowest satisfaction score prediction indicated by R^2^ values (average for the 3 scenes within each outcome variable): (1) Clarity of view, (2) Connection to the outside, (3) Color vividness, (4) Color naturalness, (5) Feeling of relaxation, (6) Reflections. While the effects of color sensitivity scores on satisfaction scores are statistically significant, they showed smaller effect. Hence, we only reported the LMM analysis results of the effect of the strongest predictor variables in the table to show the effect of scene type on view satisfaction. When predicting feeling of relaxation, reflections, and visual privacy, we found lower R^2^ values for visual acuity and contrast sensitivity scores, indicating their effects on the satisfaction scores are statistically significant but their practical utilities are quite low.

| Predictor variable | Outcome variable | Scene | ***β*** | **SE** | ***p-value*** | **R_M_^2^** | **R_C_^2^** |
| --- | --- | --- | --- | --- | --- | --- | --- |
| Visual acuity | Clarity of view | Building | -4.81 | 0.15 | <.001*** | 0.58 | 0.70 |
|  |  | Pond | -5.27 | 0.14 | <.001*** | 0.64 | 0.76 |
|  |  | People | -5.26 | 0.14 | <.001*** | 0.62 | 0.75 |
|  | Connection to the outside | Building | -4.38 | 0.14 | <.001*** | 0.53 | 0.70 |
|  |  | Pond | -4.95 | 0.14 | <.001*** | 0.59 | 0.75 |
|  |  | People | -4.44 | 0.15 | <.001*** | 0.52 | 0.70 |
|  | Color vividness | Building | -3.00 | 0.13 | <.001*** | 0.41 | 0.57 |
|  |  | Pond | -3.85 | 0.14 | <.001*** | 0.48 | 0.65 |
|  |  | People | -3.61 | 0.14 | <.001*** | 0.42 | 0.64 |
|  | Color naturalness | Building | -2.93 | 0.14 | <.001*** | 0.35 | 0.56 |
|  |  | Pond | -3.72 | 0.15 | <.001*** | 0.43 | 0.65 |
|  |  | People | -3.72 | 0.15 | <.001*** | 0.43 | 0.65 |
|  | Feeling of relaxation | Building | -2.00 | 0.15 | <.001*** | 0.18 | 0.44 |
|  |  | Pond | -2.99 | 0.15 | <.001*** | 0.34 | 0.54 |
|  |  | People | -0.01 | 0.00 | <.001*** | 0.04 | 0.32 |
|  | Reflection |  | 0.54 | 0.19 | <.001*** | 0.01 | 0.20 |
| Contrast sensitivity | Visual privacy | Building | -1.15 | 0.11 | <.001*** | 0.14 | 0.29 |
|  |  | Pond | -1.01 | 0.11 | <.001*** | 0.11 | 0.24 |
|  |  | People | -1.77 | 0.11 | <.001*** | 0.27 | 0.38 |

We found that scene type (i.e., building, pond, people) affects most satisfaction aspects (except for reflections) independently from the effect of visual acuity scores. In most cases, pond scene models show larger associations (i.e., larger *β* values) and better prediction power (i.e., larger R^2^ values) compared to building and people scenes. This indicates that a high-quality view content (e.g., the pond scene) has a stronger influence on whether view clarity will impact view satisfaction. In contrast, the effect of view clarity on satisfaction with visual privacy was stronger for the people scene. This is due to the people scene presenting a higher potential for visual privacy challenges compared to the other two scenes.

## Appendix G

“Clarity of view” and “connection to the outside” follow very similar satisfaction patterns (Appendix G-A and Appendix -B). “Color vividness” and “color naturalness” show slightly more dissatisfaction overall than “clarity of view” and “connection to the outside” (Appendix G-C and Appendix G-D). For “feeling of relaxation”, the green pond scene rates more favorably than the other two scenes for higher clarity cases, but the impact of content tends to decrease with reduction in view clarity (Appendix G-E and Appendix G-F).

#### Survey results


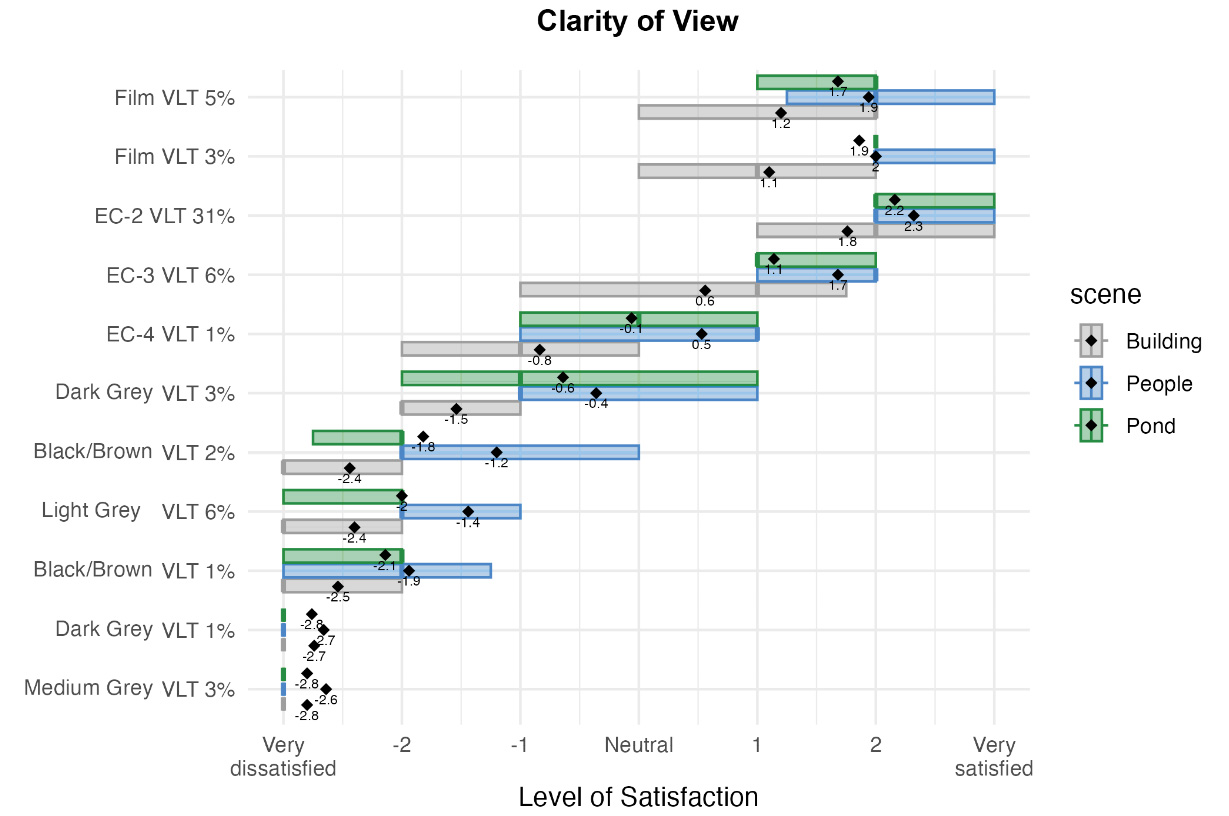


Appendix G-A. Satisfaction results for “clarity of view” (n = 50).


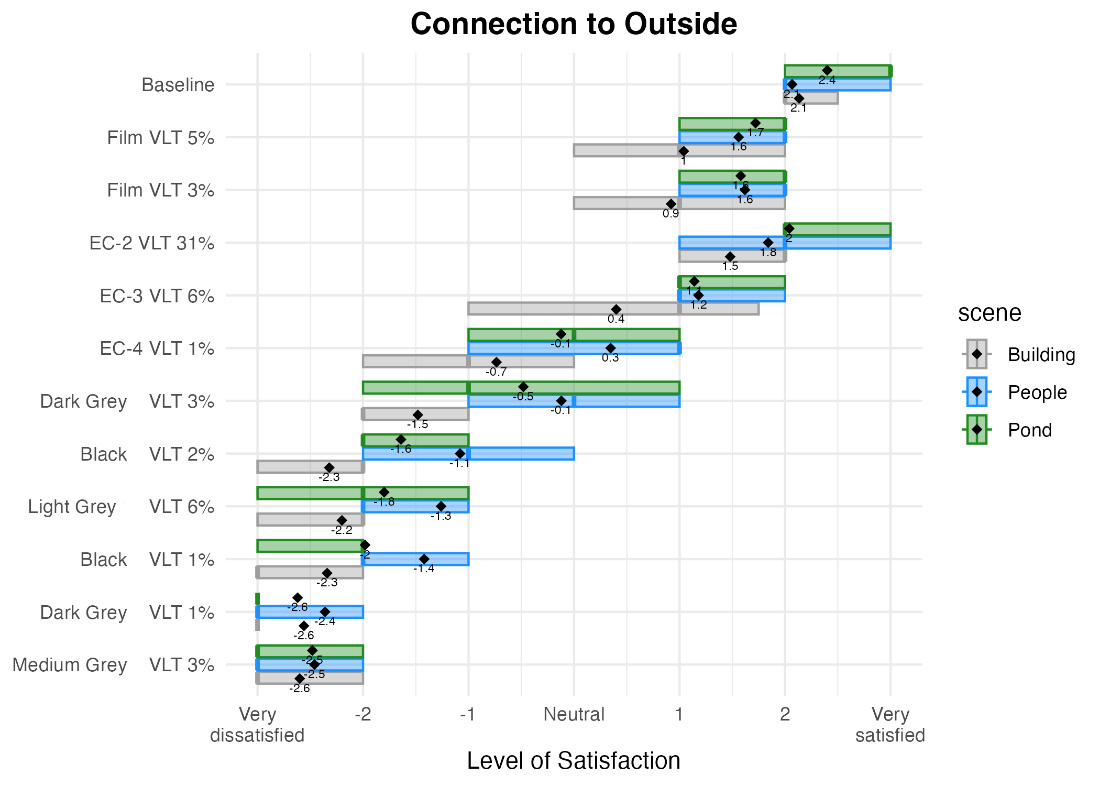


Appendix G-B. Satisfaction results for “connection to the outside” (n = 50).


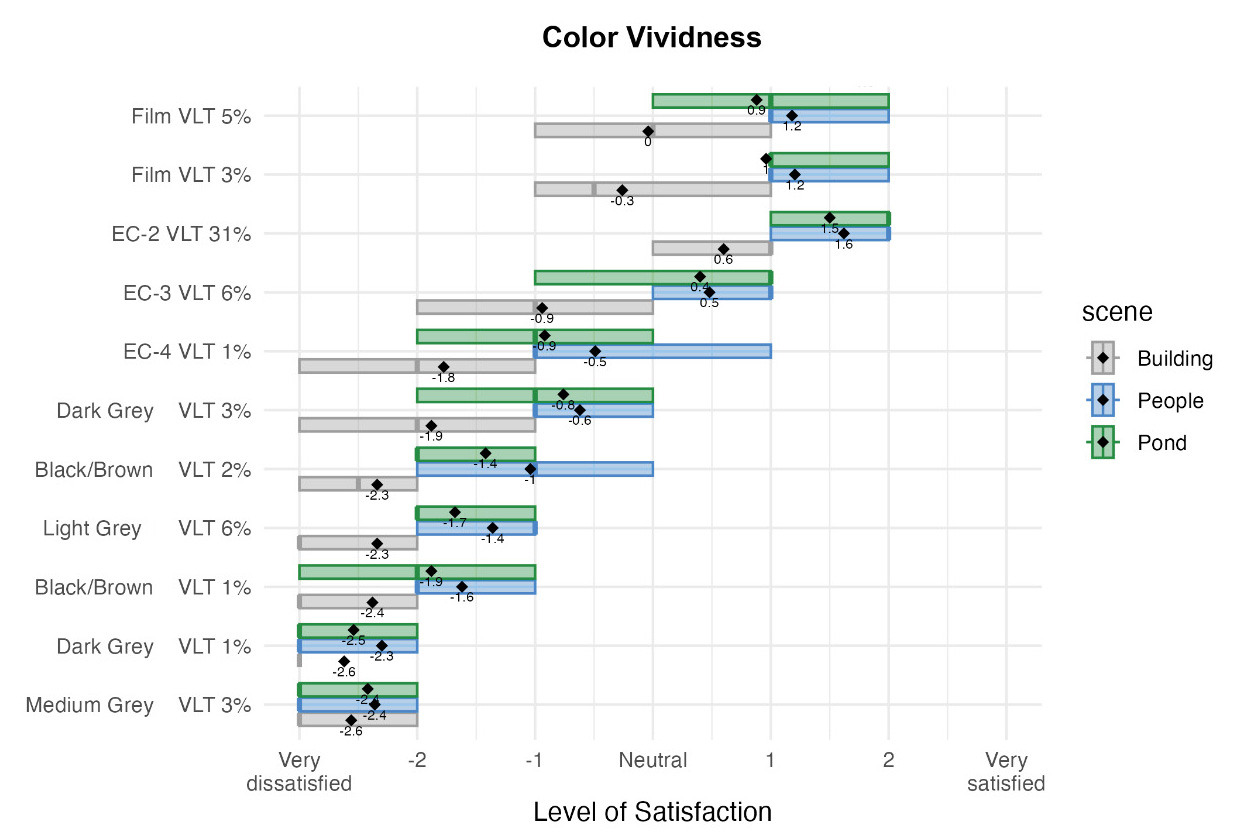


Appendix G-C. Satisfaction results for “color vividness” (n = 50).


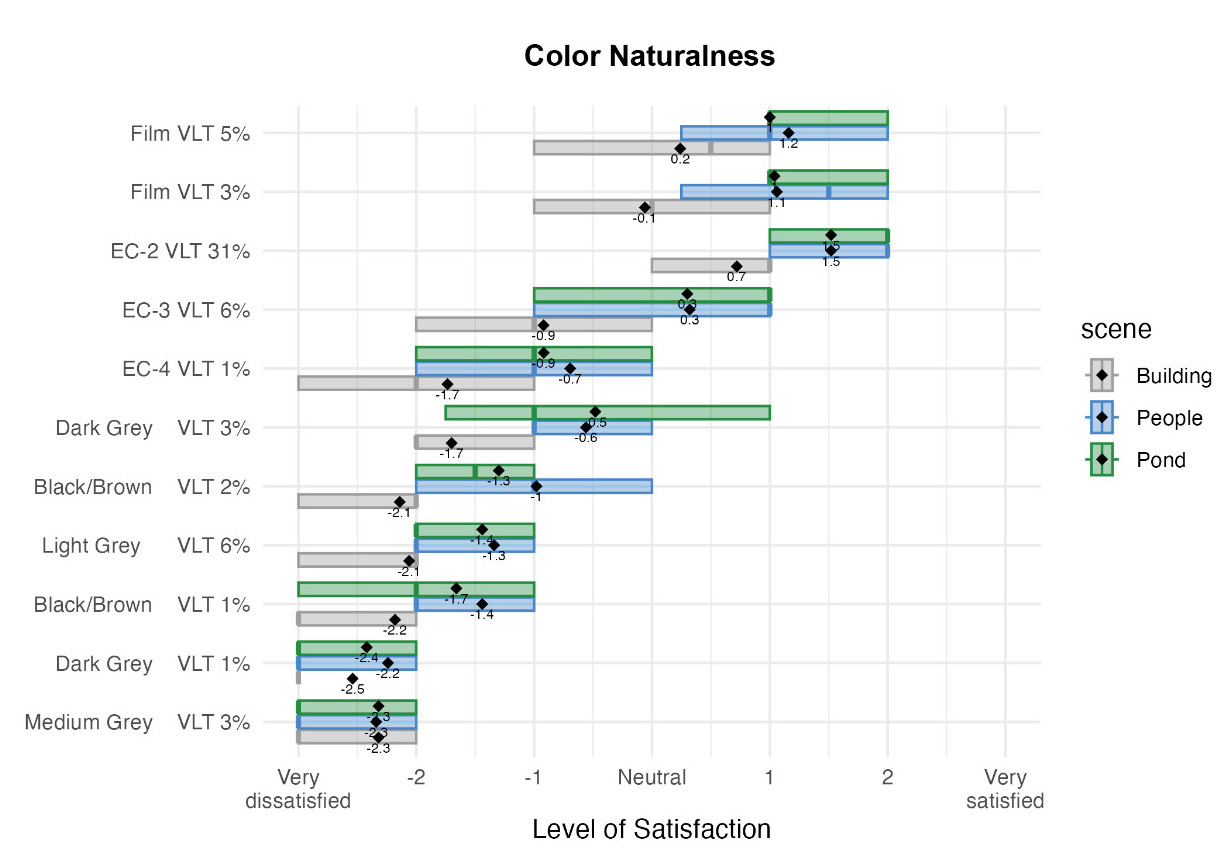


Appendix G-D. Satisfaction results for “color naturalness” (n = 50).


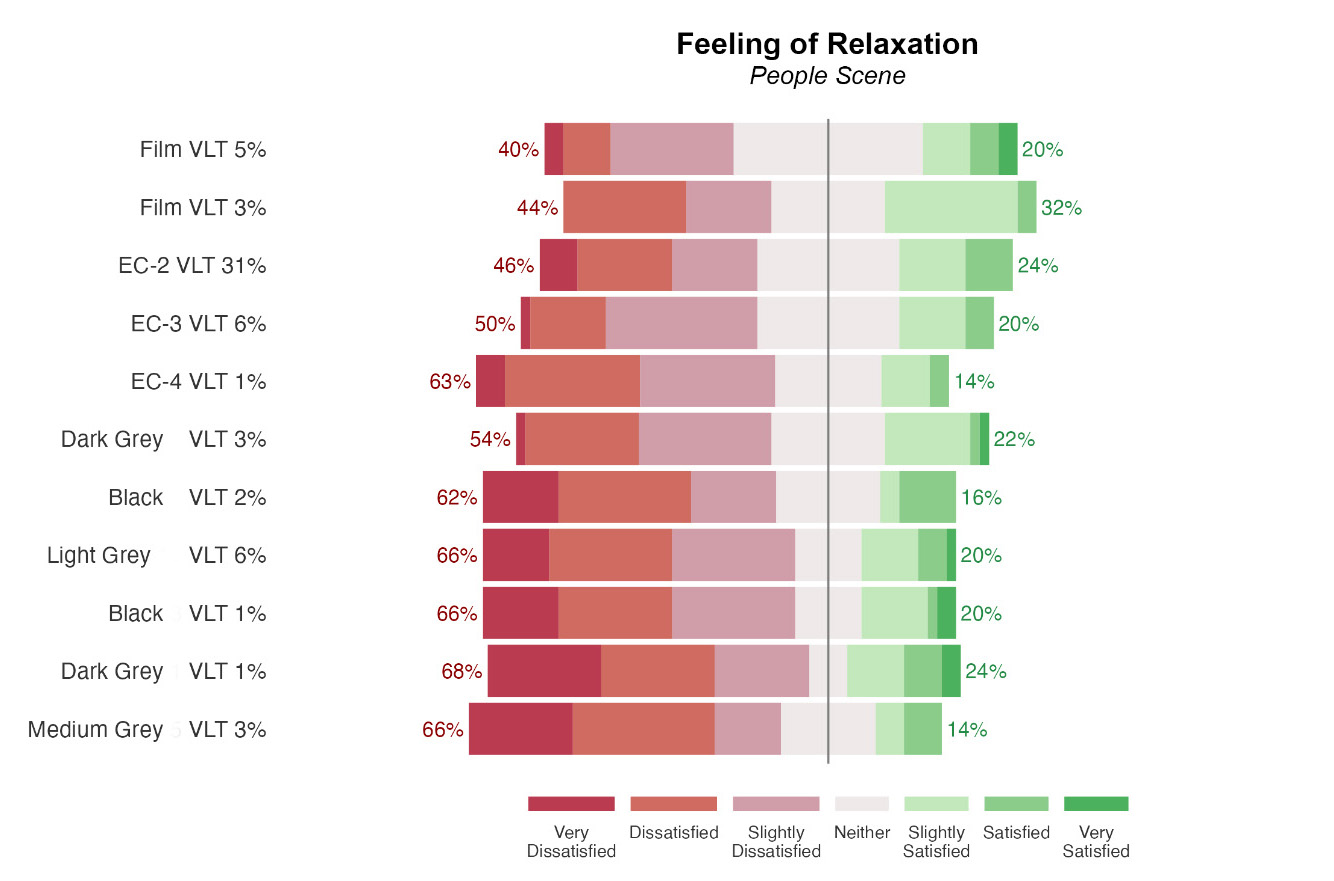


Appendix G-E. Satisfaction results for “feeling of relaxation” for the street view with people scene (n = 50).


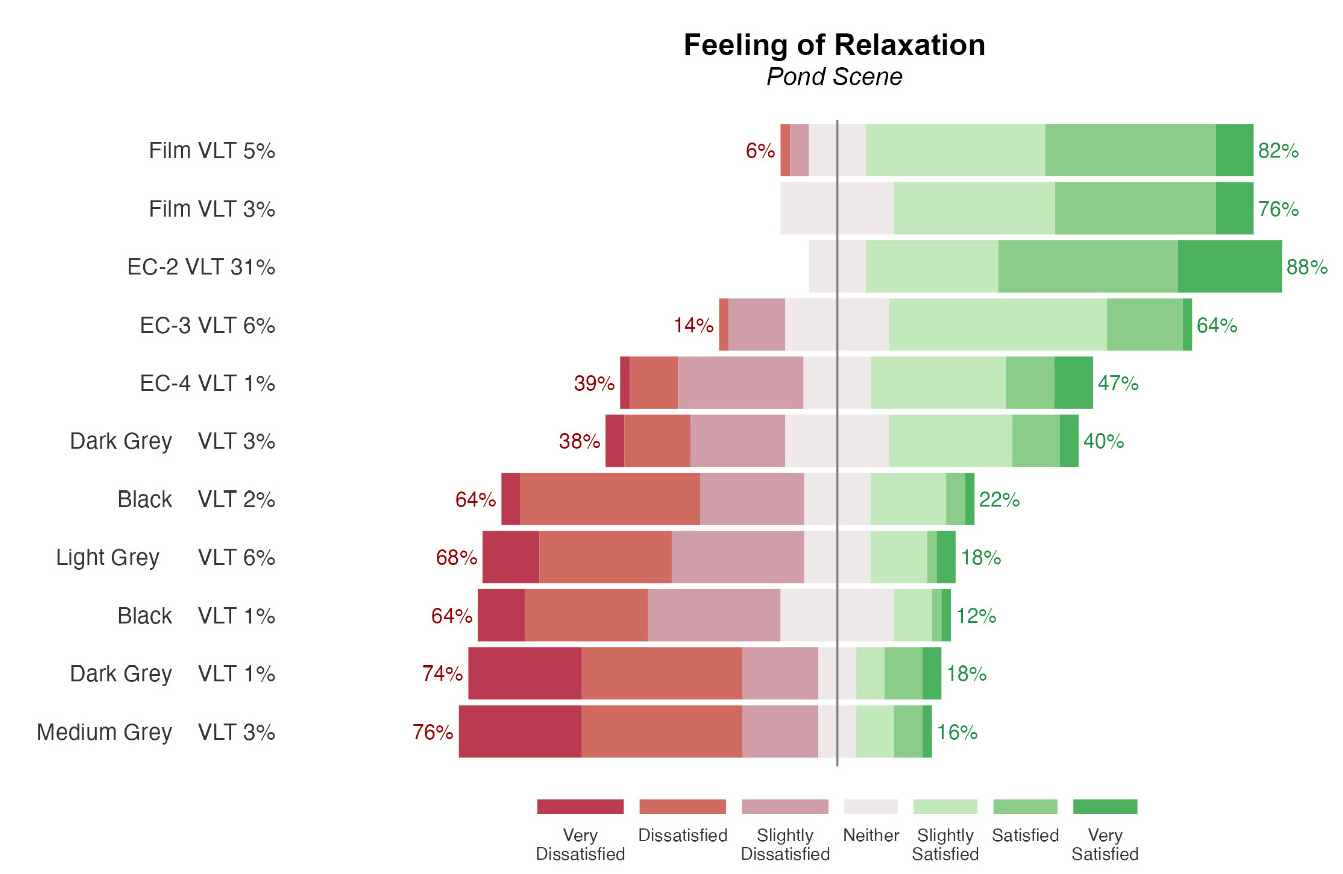


Appendix G-F. Satisfaction results for “feeling of relaxation” for the green pond scene (n = 50).

## Appendix H

#### H-A. Prediction models: the effect of optical properties of both specular and fabric shades

| Outcome variable | Predictor variable | ***a*** | ***β*** | **SE** | ***p-value*** | **R_M_^2^** | **R_C_^2^** |  |
| --- | --- | --- | --- | --- | --- | --- | --- | --- |
|  |  |  |  |  |  |  |  |  |
| Visual acuity | intercept | 0.489 |  | 0.011 | <.001*** | 0.77 | 0.81 |  |
|  | VLT |  | -0.002 | 0.000 | <.001*** |  |  |  |
|  | RS |  | 0.007 | 0.000 | <.001*** |  |  |  |
|  | Type: specular |  | - 0.658 | 0.005 | <.001*** |  |  |  |
| Contrast sensitivity | intercept | 1.164 |  |  | <.001*** | 0.73 | 0.74 |  |
|  | VLT |  | -0.001 | 0.000 | <.001*** |  |  |  |
|  | RS |  | -0.010 | 0.000 | <.001*** |  |  |  |
|  | Type: specular |  | 1.008 | 0.006 | <.001*** |  |  |  |
| Color matching | intercept | 1.164 |  | 0.011 | <.001*** | 0.33 | 0.53 |  |
|  | VLT |  | 0.002 | 0.004 | <.001*** |  |  |  |
|  | RS |  | 0.106 | 0.003 | <.001*** |  |  |  |
|  | Type: specular |  | -5.018 | 0.086 | <.001*** |  |  |  |
| Color arrangement | intercept | 44.305 |  | 1.116 | <.001*** | 0.54 | 0.61 |  |
|  | VLT |  | -0.117 | 0.034 | <.001*** |  |  |  |
|  | RS |  | 0.770 | 0.027 | <.001*** |  |  |  |
|  | Type: specular |  | -48.871 | 0.660 | <.001*** |  |  |  |
| Clarity of view  satisfaction | intercept | -1.875 |  | 0.099 | <.001*** | 0.69 | 0.80 |  |
|  | VLT |  | 0.026 | 0.002 | <.001*** |  |  |  |
|  | RS |  | -0.049 | 0.001 | <.001*** |  |  |  |
|  | Type: specular |  | 3.803 | 0.003 | <.001*** |  |  |  |
| Visual privacy  satisfaction | intercept | 1.149 |  | 0.082 | <.001*** | 0.23 | 0.39 |  |
|  | VLT |  | -0.014 | 0.002 | <.001*** |  |  |  |
|  | RS |  | 0.015 | 0.002 | <.001*** |  |  |  |
|  | Type: specular |  | -1.427 | 0.043 | <.001*** |  |  |  |
| Reflection effect | intercept | 0.69 |  | 0.20 | <.001*** |  |  |  |
| satisfaction | VLT |  | 0.02 | 0.00 | <.001*** | 0.05 | 0.27 |  |
|  | RS |  | -0.02 | 0.00 | <.001*** |  |  |  |
|  | Type: specular |  | -0.56 | 0.00 | <.001*** |  |  |  |

#### H-B. Prediction models: the effect of optical properties of specular materials

| Outcome variable | Predictor variable | ***a*** | ***β*** | **SE** | ***p-value*** | **R_M_^2^** | **R_C_^2^** |  |
| --- | --- | --- | --- | --- | --- | --- | --- | --- |
|  |  |  |  |  |  |  |  |  |
| Visual acuity | intercept | -0.12 |  | 0.02 | <.001*** | 0.20 | 0.75 |  |
|  | VLT |  | 0.02 | 0.00 | <.001*** |  |  |  |
|  | RS |  | 0.00 | 0.00 | <.001*** |  |  |  |
|  | VLT:RS |  | -0.00 | 0.00 | <.001*** |  |  |  |
| Contrast sensitivity | intercept | 2.04 |  | 0.00 | <.001*** | 0.30 | 0.42 |  |
|  | VLT |  | -0.01 | 0.00 | <.001*** |  |  |  |
|  | RS |  | -0.00 | 0.00 | <.001*** |  |  |  |
|  | VLT:RS |  | 0.00 | 0.00 | <.001*** |  |  |  |
| Color matching | intercept | 4.78 |  | 0.27 | <.001*** | 0.15 | 0.71 |  |
|  | VLT |  | 0.31 | 0.00 | <.001*** |  |  |  |
|  | RS |  | 0.10 | 0.00 | <.001*** |  |  |  |
|  | VLT:RS |  | -0.03 | 0.00 | <.001*** |  |  |  |
| Color arrangement | intercept | -0.31 |  | 0.72 | <.001*** | 0.46 | 0.73 |  |
|  | VLT |  | 1.99 | 0.04 | <.001*** |  |  |  |
|  | RS |  | 0.81 | 0.01 | <.001*** |  |  |  |
|  | VLT:RS |  | -0.22 | 0.00 | <.001*** |  |  |  |
| Clarity of view  satisfaction | intercept | 1.93 |  | 0.12 | <.001*** | 0.31 | 0.69 |  |
|  | VLT |  | -0.23 | 0.00 | <.001*** |  |  |  |
|  | RS |  | -0.08 | 0.00 | <.001*** |  |  |  |
|  | VLT:RS |  | 0.03 | 0.00 | <.001*** |  |  |  |
| Visual privacy  satisfaction | intercept | -3.94 |  | 0.60 | <.001*** | 0.05 | 0.62 |  |
|  | VLT |  | 0.07 | 0.00 | <.001*** |  |  |  |
|  | RS |  | 0.03 | 0.00 | <.001*** |  |  |  |
|  | VLT:RS |  | 0.00 | 0.00 | <.001*** |  |  |  |
| Reflection effect | intercept | 0.19 |  | 0.22 | <.001*** |  |  |  |
| satisfaction | VLT |  | -0.33 | 0.00 | <.001*** | 0.16 | 0.45 |  |
|  | RS |  | -0.07 | 0.00 | <.001*** |  |  |  |
|  | VLT:RS |  | 0.04 | 0.00 | <.001*** |  |  |  |

## Appendix I

The proposed workflow involves the following steps:

1. Test Site Set-Up

a. Establish a testing site with a window featuring clear glass (baseline) and various window shade materials. Depending on the type of shade, these materials can be installed on the exterior, interior, or within the window layers.

b. Schedule the tests at a time when the sun (or select the angle of lighting fixture) does not appear in the participants' field of view, but directional light hits the exterior side of the window.

2. Experimental Procedure

a. Request participants to read and sign a consent form.

b. Perform eligibility tests using color deficiency assessments (e.g., Ishihara number test) and visual acuity evaluations (e.g., Snellen chart) to ensure normal eye (20/20) function.

c. Conduct a practice session for human visual performance tests and view satisfaction surveys with various types of glazing and window shade materials to familiarize participants with the procedures.

d. Execute the main experiments, during which participants will complete visual acuity tests, contrast sensitivity tests, color matching tests, and satisfaction surveys for each glazing and shade material case. Ensure the order of materials is randomized for each participant.

3. Predictive Framework Establishment

a. Apply Linear Mixed Model (LMM) analysis or other relevant statistical modeling methods depending on data to identify primary human visual performance factors that predict view satisfaction.

b. Test various combinations of primary variables to develop the final prediction model with the highest predictive power (e.g., R²), while avoiding multicollinearity.
